# Intolerant baboons avoid observer proximity, creating biased inter-individual association patterns

**DOI:** 10.1038/s41598-022-12312-3

**Published:** 2022-05-16

**Authors:** Andrew T. L. Allan, Amy F. White, Russell A. Hill

**Affiliations:** 1 Department of Anthropology, Durham University, Dawson Building, South Road, Durham, DH1 3LE UK; 2 Primate and Predator Project, Lajuma Research Centre, PO Box 522, Louis Trichardt, 0920 South Africa; 3 Department of Zoology, University of Venda, Private Bag X5050, Thohoyandou, 0950 South Africa

**Keywords:** Behavioural ecology, Animal behaviour

## Abstract

Social network analysis is an increasingly popular tool for behavioural ecologists exploring the social organisation of animal populations. Such analyses require data on inter-individual association patterns, which in wild populations are often collected using direct observations of habituated animals. This assumes observers have no influence on animal behaviour; however, our previous work showed that individuals in a habituated group of chacma baboons (*Papio ursinus griseipes*) displayed consistent and individually distinct responses to observer approaches. We explored the implications of our previous findings by measuring the inter-individual association patterns of the same group of chacma baboons at different observer distances. We found a strong positive association between individual tolerance levels (towards observers) and how often an animal appeared as a neighbour to focal animals when observers were nearer, and a neutral relationship between the same variables when the observer was further away. Additionally, association matrices constructed from different observation distances were not comparable within any proximity buffer, and neither were the individual network metrics generated from these matrices. This appears to be the first empirical evidence that observer presence and behaviour can influence the association patterns of habituated animals and thus have potentially significant impacts on measured social networks.

## Introduction

In behavioural ecology research, habituation processes have been used to reduce the fear animals have towards humans, allowing behavioural data to be collected directly^1^. An expansive body of literature suggests this process is robust to potential issues. However, direct observations of habituated animals contain two implicit assumptions that are rarely acknowledged or tested empirically. Firstly, that observers have a neutral effect on study animals, and secondly, that all study animals are habituated equally^2^. We found evidence that neither assumption was valid in a habituated group of chacma baboons, with observers viewed as equivalent to a high-level social threat and while tolerance of observers (the distances at which individuals visually oriented towards and displaced away from approaching observers) was consistent within individuals it was distinct between them^2^. These findings open up a range of new questions regarding how the presence of variation in observer tolerance impacts the behaviour and ecology of habituated animals.

Observer neutrality has only received minor discussion in species often exposed to direct observations such as primates^1,3,4^ and meerkats^5^, although observer effects on the behaviour of habituated animals have been reported elsewhere. Habituated bat-eared foxes (*Octocyon megalotis*) were found to increase vigilance during the beginning phases of focal observations^6^ suggesting that habituation had not led to complete observer neutrality and some fear of humans remained. In reef fishes, cleaning interactions between individuals were found to be more frequent when divers (observers) were absent, despite a long history of diver presence in the area^7^. If observers have a similar effect on other habituated animals and inter-individual differences in tolerance remain once habituation processes are deemed completed, then observer-governed effects could have fundamentally biased our understanding of a range of behaviours across the animal kingdom.

Characterising the social systems of animals is often attempted through sampling inter-individual association patterns, such as affiliative and agonistic interactions, or spatial proximity between individuals^8^. Over the last decade, social network analysis has become an increasingly popular tool for visualising and analysing these types of data^9^ and has been used to explore a number of broad themes, including the fitness consequences of sociality, identifying individual social roles within groups, and mapping disease transmission in wild populations^10,11^. Sampling inter-individual association patterns directly is reliant upon the assumption that observers do not impact on social interactions, yet observer effects appear mostly overlooked. Recent research has shown that humans can influence typical patterns of social relationships in wild primates^12^, whilst there is concern amongst researchers that observers are unlikely to ever become a neutral stimulus and the presence of unhabituated behaviours may undermine the validity of data^13^. The presence of variation in observer tolerance (i.e., our previous study^2^) would also indicate that both observer presence and behaviour (e.g., distance to animals, observer movement etc.) could lead to observer-governed phenotypic assortment, with intolerant animals adjusting their spatial position to avoid the observer. Such an effect would undoubtedly bias inter-individual association patterns as intolerant phenotypes may be under-sampled relative to tolerant animals.

Here we explore the implications of individual variation in tolerance to observers and test whether observer proximity can influence inter-individual association patterns in a group of habituated chacma baboons. Baboon association patterns have been sampled using a range of proximity measures and affiliative interactions^14–16^, however, different sampling methods can produce different networks^14^. Despite this, there has been little discussion of whether association patterns can be affected by observer presence and behaviour during direct observations. To address this, we recorded the proximity associations of all group members when the observer collected data in close proximity compared to more distant observations. We then explored whether the interaction between individual tolerance (of each baboon towards the observer) and observer distance influenced how often each individual was sampled in proximity of focal animals, and whether the hypothesized trends were consistent across different years. If intolerant individuals avoided observers, we predicted that intolerant phenotypes would be sampled (as neighbours to focal animals) as often as other individuals when the observer was further away but be sampled less often when the observer was close.

To further illustrate the implications of individual variation in tolerance to observers using more traditional network analysis approaches, we constructed dyadic proximity association matrices (weighted and symmetric) for observations collected when the observer was close versus distant (separately within each year and proximity buffer) and compared association scores between these paired matrices. We subsequently explored whether individuals exhibited similar local positions (i.e., degree, betweenness, closeness) in the networks produced from these association matrices. As with our prior analysis, if intolerant animals avoided observers, then we predicted there would be little evidence supporting positive correlations between the paired matrices or the individual network metrics. Collectively, if our predictions were confirmed, these findings would have important implications for future studies using direct observations to collect data on inter-individual association patterns.

## Methods

All research methods included in this study were performed in accordance with the relevant guidelines and regulations, under ZA/LP/81996 research permit, with ethical approval from the Animal Welfare Ethical Review Board (AWERB) at Durham University. The authors confirm the study was carried out in compliance with ARRIVE guidelines.

All inter-individual association data was collected between June 2018 and June 2019 on a wild habituated group of Afro-montane chacma baboons in the western Soutpansberg Mountains, South Africa (central coordinates S29.44031°, E23.02217°) (for study site description see^2^). The study group was habituated circa 2005 and was the focus of intermittent research attention until 2014. The study area experienced long-term anthropogenic activities (local farming, forestry, and residences) prior to 2005, as such, consistent interactions with humans have been ongoing with this population for some time. From 2007 onwards numerous researchers were able to collect expansive datasets on the study group (e.g. Refs.^17,18^), indicating that habituation was at a typical level found elsewhere (also validated by AA and RH, who had researched chacma baboons elsewhere). From 2014 the group received full day (dawn until dusk) follows 3–4 days a week, with occasional gaps of up to 5 weeks in duration. These gaps did not appear to effect habituation levels, likely due to the presence of other researchers at the field site who always tried to act benignly when encountering the habituated group. The follow schedule was designed so that the study group retained as much of their natural interactions with predators as possible by ensuring the baboons spent significant time without observers who may influence the frequency and nature of predator–prey interactions^19^.

The study site was located in a private nature reserve and the study group was not hunted during observation gaps or engaged in any conflict with humans, other than occasionally being scared (chasing, yelling, throwing stones etc.) from a small plantation by local workers, usually resulting in alarm barks and fleeing responses. However, the study group appeared adept at recognising the differences between researchers and these threats^20^. The majority of the study group’s home-range typically overlapped with the core area of the Lajuma Research Centre, and as a result, interactions with staff living in the area, unfamiliar researchers, and tourists were frequent. However, the baboons had not engaged in ‘raiding’ residences, threatening humans, or any other potentially negative symptom of habituation before the end of this study.

### Sampling methodology for proximity associations

30-s focal sampling was used to collect proximity associations between all group members (excluding infants). All data was collected between June and December 2018 and January and June 2019; the majority of 2018s data was collected during the wet season, whilst most of 2019s data was collected during the dry season. To account for time of day, each day was split into four time-periods that were seasonally adjusted ensuring each period accounted for 25% of the current day length. A randomly ordered list of individuals was produced for each day, the first individual identified from the top 15 (approx. 20% of group size) individuals on the list was sampled immediately. Individuals could only be sampled once per time period per day, and a maximum of twice total per day. All individuals received at least 14 focal observations per time period (56 total) across the study period (see below for how we handled uneven sampling for some individuals). A video camera was used by AA (the only observer to collect this data) to record all focal observations (Panasonic HC-W580 Camcorder). At the end of the 30-s focal observation the identities of all neighbouring conspecifics within 5 m, 2.5 m, 1 m, and touching the focal animal were recorded (audibly by AA). We chose the end of the focal observation to record this data as this was most likely to reflect the conditions during the focal, i.e., the observer had been in proximity for at least 30 s.

Neighbour information was extracted from video footage and entered manually by AA and AW. Data was split into separate years to reflect an observation gap of several weeks and to understand whether there was consistency in the hypothesized effects through time and to reflect underlying differences in environmental conditions during the two study periods; during the dry season fruits and seeds are scarce and day lengths are several hours shorter than in the wet season such that day journey lengths are often shorter in the dry season and animals are much more sedentary which could impact inter-individual spacings. In 2018 each individual was sampled between 28 and 30 times; 28 focals were randomly selected from each individual to make sampling even. For 2019 there were between 25 and 27 focals per individual; 25 samples of each individual were randomly selected. Observations were undertaken at a range of distances. For both years the median end observer distance was 4.5 m; data was thus split into close focal observations of less than or equal to 4.5 m (2018: n = 918, 2019: n = 809), and observations greater than 4.5 m (2018: n = 902 2019: n = 816). See supporting information Table S1 for summary statistics of the observation distances of each individual.

We did not make any attempt to record our focal data evenly across the various habitats at our field site (see Supporting information text S1 for complete habitat descriptions) as our previous research indicated there was little difference in general spatial cohesion/inter-individual proximity patterns across these habitats (see Supporting information text S2 and Table S2). As a result, we considered it unlikely that there were fundamental differences in inter-individual association patterns across habitats, or that observers struggled to reliably detect or identify neighbours in dense habitats. We do acknowledge, however, that there will always be an element of bias with such methods, as observations were avoided, aborted, or excluded if visual obstructions (e.g., cliffs, rocks, walls, buildings, very dense vegetation etc.) prohibited accurate assessments; the observations used in the current study are from occasions when these factors were not an issue.

During this study the group contained between 85 and 92 individuals. Age-sex class was defined according to secondary sexual characteristics (e.g., testes descending/enlarging, sexual swelling, canine eruption) and changes in pelage throughout juvenile development (see Supporting information text S3 for full descriptions). All 65 non-infant individuals that were present during 2017 (when displacement tolerances were calculated) and still remaining in the group by the end of 2019 were used in this study (4 individuals from the prior FID study were no longer present). There were a high number of births between 2018 and 2019, but none were independent by the time either of our sampling periods begun in 2018 or 2019. There was no immigration of foreign individuals, but two individuals disappeared, both during the 2018 focal sampling period. As a result, we had a very consistent pool of individuals to sample from during this study. We removed all data associated with the two individuals who disappeared as their occurrences as neighbours would have been poorly sampled (due to missing more than half the study) relative to the rest of the group which would have led to statistical biases^21^.

### Flight initiation distance procedure

Individual displacement tolerance estimates were previously quantified in our previous research^2^ using a flight initiation distance (FID) procedure^22^ that was completed between October 2017 and April 2018, prior and independent to the commencement of proximity association focal sampling in June 2018. Individual baboons were approached by an observer, and the distance at which the animal displaced away from the observer measured (see Supporting information Table S2 for summary statistics). This procedure was repeated 24 times for each individual baboon, with approaches spread evenly across two observers differing in familiarity. At the beginning of each approach we also recorded several behavioural, social, and environmental factors that could have hypothetically influenced an individual’s FID^2^ including whether the animal was engaged (e.g., digging or grooming) or not engaged (e.g., resting, chewing food, being groomed), habitat type (open/closed: see Supporting text S1), whether the animal was on the ground or sat on a low branch or rock within 50 cm of the ground, the number of conspecifics within 5 m of the focal animal, and whether there had been any external events within the preceding 5 min (e.g., alarm calls, aggressions, encountering another group or predator). During the approach, we also recorded the visual orientation distance (the distance at which the focal animal directed its line of vision towards the head of the approaching observer) and whether one of the focal animal’s neighbours had displaced/fled before the focal animal. Although all but neighbour flee first and external events showed some importance for predicting looking (see Table S4), FID was found to be distinct amongst individuals and repeatable within each individual, evidence that displacement tolerance may be an individual level trait^2^. Full details of methods, statistical analysis, and results (including comparison to the original model) for this updated model are in Supporting information text S4, with model summary results for the previous and updated models in Tables S3 and S4.

The notion of an observer approaching a habituated primate may be considered atypical or likely to result in habituation/sensitization effects or agonistic behaviours being directed towards the approaching observers. However, our previous study^2^ showed that almost all approaches resulted in the animal passively relocating (98.85%), a very benign response identical to the behaviours of subordinate baboons displacing away from dominant conspecifics. This suggests that in this group, observers may be considered equivalent to a high-level social threat^2^. Throughout observation periods on habituated animals, observers are likely to approach or displace animals either incidentally or accidentally multiple times throughout the day, especially during lengthy focal observations. As such, the approach methodology is unlikely to represent a stimulus outside of the norm for our study animals. This may explain why displacement responses were so passive and why there was no evidence of habituation or sensitization effects across the group or individually through a range of temporal periods^2^ or after life-threatening events^20^. As a result, our situation was possible without risk of causing stress or anxiety in the study subjects, eliciting agonistic behaviours towards observers, or interfering with their prior habituation levels.

### Statistical analysis

#### Influence of tolerance and observer distance on inter-individual association patterns

##### Quantifying displacement tolerance

To quantify displacement tolerance towards observers we extracted the individual conditional modes from the updated FID model using the *ranef* function in *brms.* Conditional modes are often referred to as Best Linear Unbiased Predictors (BLUPs) and are the difference between the predicted mean population-level response for a given set of treatments (i.e., population-level effects) and the predicted responses for each individual, and therefore infer the extent to which each individual differs from the population mean. The conditional modes and their associated standard deviations can be found in supporting information Table S5.

To validate that the conditional modes from the updated model were both representative of the individual’s flight responses and in line with the estimates produced from our previous study^2^ we performed additional tests. Firstly, we performed a Pearson’s correlation between the conditional modes from the updated model and the conditional modes from the previous article. Individual tolerance estimates were consistent (r(63) = 0.915, *p* < 0.001) despite the changes in model structure from^2^. Additionally, the intraclass correlation coefficient remained almost identical (updated individual identity ICC: 0.60; highest density intervals (HDI) for posterior samples at 95% intervals, 0.50, 0.70) to the findings reported in our previous research^2^ (ICC, 0.65; HDI, 0.56, 0.74). Finally, we also explored the correlation between the conditional modes of the updated FID model and each individual’s mean and median FIDs as raw measures have also been used to estimate individual traits, e.g., time spent inspecting a novel item for boldness^23^ and proportion of days trapped for trappability^24^. We found high correlation between the conditional modes and each individual’s mean (r(63) = 0.861, *p* < 0.001) and median (r(63) = 0.847, *p* < 0.001)) FID values.

To ensure the data collected from the 2017/2018 FID approaches were applicable to focal data collected throughout 2018 and 2019 we tested a further 15 individuals (approximately 25% of the group) beginning two weeks after the proximity focals were completed (late June 2019). We tested the effect of year on individual tolerance estimates and found them to be consistent across years (see supporting information text S5 for details). Thus, we considered the flight initiation distance data collected during 2017/18^2^ to be applicable to our current study.

#### Mixed model analysis investigating the interaction between individual tolerance and observer distance on individual occurrences as a neighbour

The inter-individual association data were coded as a count of how often each individual occurred as a neighbour during the focal observations of the remaining (n-1) group members. Each individual had separate counts for each observer distance (i.e., over/under 4.5 m) within each proximity buffer (i.e., touch, 1 m, 2.5 m, and 5 m), separately for each year (i.e., 2018 and 2019). These counts were the response variables in four generalised linear mixed effects models (separate models for each proximity buffer). In all models, the count of an individual’s occurrences as a neighbour was predicted by the interaction between observer distance, individual tolerance estimate (conditional modes from updated FID model), and year. Tolerance was on the spectrum whereby low/negative values indicated low tolerance and high (positive) values indicated high tolerance. We specified that all combinations of the fixed effects and their two-way interactions were also modelled.

Although using conditional modes without their associated error is not ideal (see^25^ for discussion), the alternative approaches were not viable for our data. Firstly, measurement error models produced divergent transitions with very low effective sample sizes rendering inference from the models unreliable. Secondly, multivariate approaches were exceptionally challenging to initialise and fit; in our case as the bivariate model with dual response variables of FID and individual occurrences as neighbour drew from two datasets derived from independent methodologies requiring distinct model structures and response families. As a result, despite our conditional modes losing the error around the point estimates, our models using these estimates were the most robust we could fit and draw inference from. Previous research has also consistently used this approach (without the associated error) in personality studies as response variables^26,27^, population-level/fixed effects^28,29^, or as variables in correlations and principal component analysis^30^. However, we do acknowledge that losing the associated error from the conditional modes may contribute to anticonservative estimates in the models analysing occurrence patterns.

For all models we specified the interaction between tolerance and observer distance as a random slope over individual identity, with correlated intercepts. As with the population-level effects, we also allowed the interaction to model each covariate within the interaction as separate slopes over individual identity, whilst year was also included as a separate random slope over individual identity to ensure we estimated the within individual and between individual differences (in occurrences as a neighbour) due to sampling year. We did not model this as a 3-way interaction due to model performance issues; however, the tolerance and observer distance interaction was vital to ensure we effectively estimated the differences (in occurrences as a neighbour) due to between individual tolerance differences, the individual slopes of observation distance, and the between individual differences due to the interaction between tolerance and observation distance.

We did not centre or scale any variables as year and observer distance were categorical and tolerance represented each individual’s mean difference to the population mean. We also calculated how many focal samples it was possible for each individual to appear in (as a neighbour) as each individual was focal sampled differentially. For example, there were 888 focal samples (of the n-1 other individuals) that individual ARL could have occurred as neighbour in 2018 with the observer over 4.5 m away, whilst there were 886 for individual ATH for the same year and observer distance due to ATH receiving two more focal observations than ARL in these contexts. We included the natural log of this variable as an offset term to account for this differential sampling. All models were fit with a Bayesian procedure using the *brm* function^31^ in the R software^32^. Each model was run for four Hamiltonian Markov chains for 15,000 iterations, with warmup iterations set to 5000, and adapt_delta set to 0.95 all these parameters were set higher than default. A Poisson response distribution was defined, with default link functions. Default improper flat priors were assigned to all population-level effects and Student t default priors (df = 3, mean = 0, scaling factor = 2.5) assigned to the remaining model components^31^. However, in the case of the standard deviations of group-level (i.e., random) effects these parameters are constrained to be positive and therefore half Student-t priors were implemented.

Model fits were assessed via graphical checks, firstly by examining trace plots to ensure convergence/good mixing of multiple chains, and secondly, by comparing our observed data to data simulated from the posterior predictive distribution of our models using the pp_check and pp_stat functions (*brms* and *bayesplot* packages). In each case the simulated data from our models generated data that captured the vast majority of values in our observed response distribution with no signs of dispersion issues^31,33^. To further assess that there were no dispersion issues, we followed^34^ and fit each proximity buffer model (i.e., 5 m, 2.5 m, 1 m, and Touch response variables) with a negative binomial response distribution and compared each model to their respective Poisson models using leave-one-out cross-validation (LOO-CV). Although the models performed similarly, in all cases the Poisson models produced higher expected log-predictive densities than the negative binomial models, indicating higher predictive accuracy and therefore confirming that there were no dispersion issues with any of our Poisson models^34^.

The outputs of each model also showed that all Rhat values were equal to 1, indicating accuracy of the response variable with regard to the Poisson response distribution and excellent chain convergence. In addition, the bulk effective sample sizes (ESS) were considerably above the minimum advised threshold (100 times the number of chains), with tail ESS also being well sampled. Together these factors highlight that the model produced high estimation accuracy, including at the tails of the distribution^31^. Finally, we checked for multicollinearity between variables using the *check_collinearity* function from the *Performance* package^35^, and all predictors produced variance inflation factors of less than 4, strong evidence that multicollinearity was not an issue.

Relying purely on model estimates and 95% credible intervals for inference can be subjective and overlooks the extent to which the posterior is equivalent to zero^36^. Therefore, to ensure as much information about the posterior was included in inference, we additionally calculated the 89% Highest Density Interval (HDI) of the posterior distribution, the percentage of the posterior distribution within the region of practical equivalence (ROPE), and the probability of direction (pd) for each population-level (i.e., fixed) effect within each model. The HDI reveals the upper and lower parameter values of the posterior based on all values within the 89% range, these points therefore have a higher probability density than those outside the 89% range^37^. The ROPE is the region that equates to the null hypothesis, although there are no set rules for defining this interval as it relates to the variables being analysed^37^. In our case the response variable was a count and so we defined the area around 0 that equated to the ‘null’ as − 0.1 to 0.1, and this was additionally validated using the *rope_range* function from *bayestestR*^38^*.* We therefore computed the proportion of the 89% HDI of the posterior distribution within the − 0.1 to 0.1 ROPE range. The pd variable is an index for inspecting whether each fixed effect has directionality (i.e., is positive or negative); pd always ranges from a minimum of 50% (i.e., equal distribution of positive and negative posterior values) to 100% (i.e., all posterior values are either positive or negative)^39^. It has been shown that pd has a 1:1 correspondence with p-values calculated using frequentist methods^39^.

We also calculated the conditional Bayesian R^2^ estimates for each model using the *loo_R2* function from the *performance* package ^40,41^. In ordinary least squares (OLS) and maximum likelihood estimation regressions (MLE) the R^2^ value is the variance of the predicted values divided by the variance of the data. In Bayesian analysis however, the variance of the predictions can be greater than the variance of the data. To address this issue, a Bayesian R^2^ has been proposed^40^, in which the R^2^ estimate is the variance of the modelled predicted means divided by the sum of variance of the modelled predictive means and the modelled residual variance. The Bayesian R^2^ value is therefore an estimate of the proportion of variance explained for future observations predicted using a given model, i.e., it is data-driven^40^.

### Implications to proximity association matrices and network analyses

#### Constructing association matrices collected at different observation distances

Symmetric, weighted (i.e., valued) dyadic association matrices were constructed for proximity data collected at different observation distances separately for each proximity buffer across both years, resulting in a total of 16 matrices. Matrices were symmetric because connections between individuals were derived from undirected scans of the proximity of neighbouring baboons to a focal animal^14^. We initially calculated the strength of connection between each dyad as the sum of how often each individual occurred as a neighbour of one another. The median (plus min and max) focal samples for each dyad were as follows: 2018 _within 4.5m_: 26 (2–42), 2018 _over 4.5m_ 29 (2–51), 2019 _within 4.5m_ 27 (5–55), and 2019 _over 4.5m_: 24 (8–50). As individuals were not sampled evenly across both observation distances (within/over 4.5 m), we converted this value to a proportion by dividing the connection strength by the sum of both individual’s (in each dyad) sampling effort within each observation distance. This gave each potential dyad exactly two connections of equal values (i.e., symmetrical): the proportion of their combined focal observations they occurred in proximity to one another for each observation distance. This was done separately for each proximity buffer within each year.

### Comparing association matrices collected at different observation distances

To determine whether there were significant differences between association matrices collected when the observer was within 4.5 m versus beyond 4.5 m, we ran mantel tests between the matrices of each proximity buffer, within each year, using the *mantel* function from the *vegan* package^42^ with permutations set to 999 and Spearman’s rank as the correlation method. Mantel tests perform correlation tests between pairs of matrices and produce a standardised Mantel test statistic ranging from − 1 to 1 that infers the strength of the relationship between paired points from each weighted/directed matrix.

### Investigating whether individuals exhibit similar local positions in networks produced from different observation distances

We used the R package igraph^43^ to generate a network for each of the aforementioned matrices and to calculate the individual network metrics. We assessed each individual’s centrality on three levels: (1) degree—the total of an individual’s out- and in-degree connections, (2) closeness—the distance required for an individual to access the remaining individuals, and (3) betweenness—the number of shortest paths (between dyads) going through an individual. As proximity associations were considered undirected, we utilised only undirected metrics. As the shortest paths were weighted they were interpreted as distances^43^. We used non-parametric Spearman’s rank correlations to investigate whether individuals held similar rank order positions for degree, closeness, and betweenness across networks produced from different observation distances. This was done separately for each proximity buffer within each year.

## Results

### Influence of tolerance and observer distance on inter-individual association patterns

**Figure 1:**
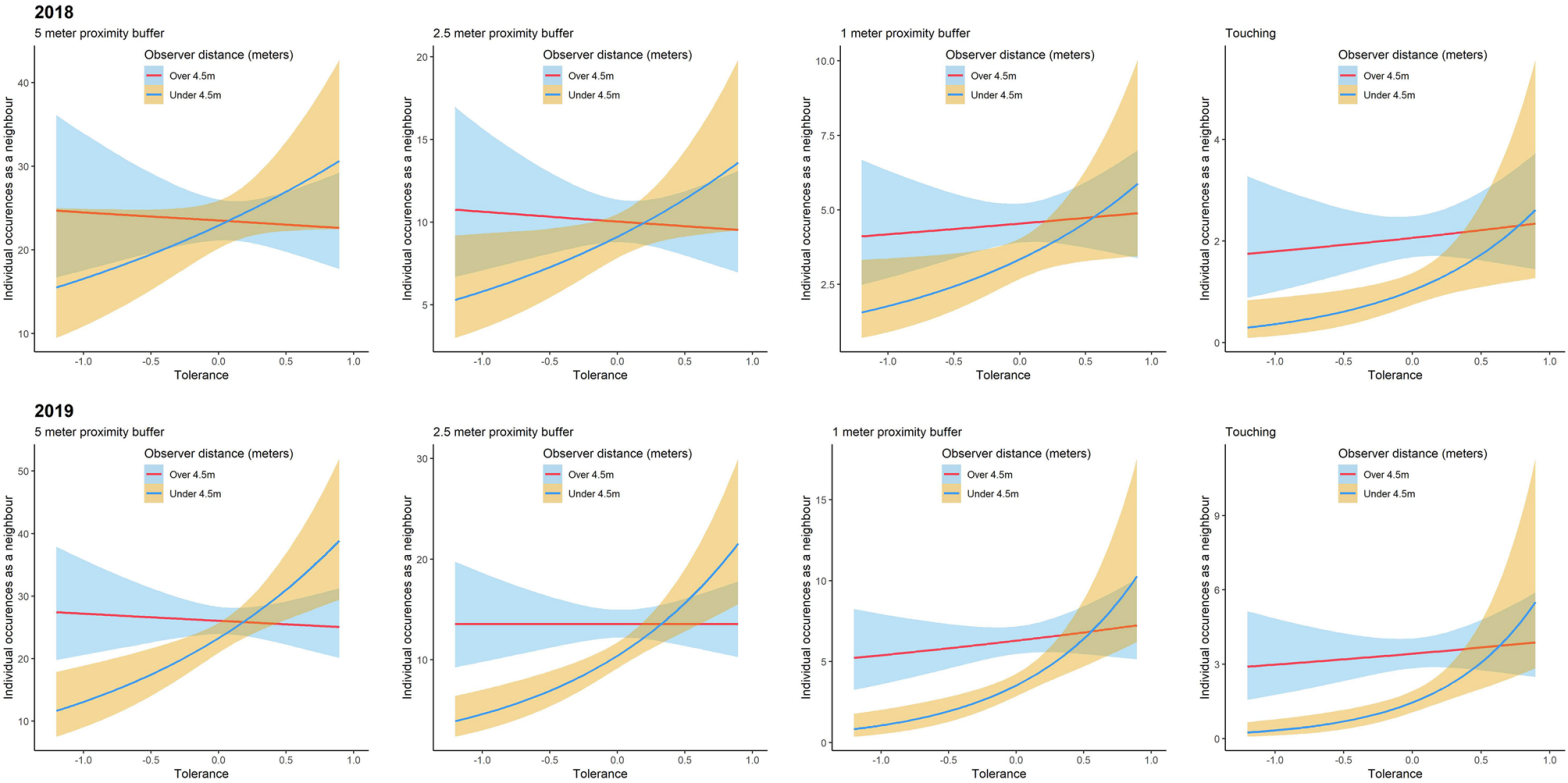
Conditional effects plots of model results predicting the effect of the interaction between individual displacement tolerance (high values indicate highly tolerant animals) and observer distance on the number of occurrences each individual has as a neighbour for the remaining group members for 2018 and 2019. The mean was used as the central tendency, with the shaded areas displaying the relevant credible intervals (2.5 and 97.5% quantiles).

The population-level effects calculated from the mixed model analysis (see Table 1) suggest strong evidence that the 2-way interaction between tolerance and observer distance influenced the population-level mean for occurrences as a neighbour. Conditional effects plots (see Fig. 1) display the predicted population-level means for number of occurrences as a neighbour conditioned on the interaction between tolerance and observer distance; for each year; highlighting a consistent (across years and proximity buffers) positive relationship (when the observer was at less than 4.5 m) between tolerance and the population-level mean for occurrences as a neighbour. Interestingly, this effect is neutralised when data is collected from over 4.5 m, i.e., the population-level mean remained relatively constant across the tolerance spectrum.

**Table 1:** Model summary results for population-level effects for all four models, columns represent the data recorded from each proximity buffer.

	5 m Buffer	2.5 m Buffer	1 m Buffer	Touch Buffer
Bayesian R^2^	0.51 (0.05)	0.37 (0.05)	0.22 (0.05)	0.23 (0.06)
Intercept	*− ****3.59 (****− ****3.67, ****− ****3.5)***	*− ****4.44 (****− ****4.54, ****− ****4.33)***	*− ****5.23 (****− ****5.35, ****− ****5.12)***	*− ****6.02 (****− ****6.18, ****− ****5.86)***
Observer Distance (Under 4.5 m)	− 0.03 (− 0.1, 0.05)	− 0.1 (− 0.21, 0.01)	*− ****0.31 (****− ****0.49, ****− ****0.13)***	*− ****0.7 (****− ****0.99, ****− ****0.41)***
Tolerance	− 0.04 (− 0.27, 0.19)	− 0.06 (− 0.34, 0.23)	0.08 (− 0.23, 0.39)	0.14 (− 0.27, 0.55)
Year (2019)	0.1 (0.03, 0.17)*	***0.3 (0.2, 0.39)***	***0.33 (0.2, 0.46)***	***0.51 (0.33, 0.68)***
Observer Distance (Under 4.5 m) : Tolerance	***0.37 (0.14, 0.59)***	***0.51 (0.19, 0.81)***	**0.55 (0.05, 1.07)**^+^	**0.91 (0.15, 1.66)**
Observer Distance (Under 4.5 m) : Year (2019)	− 0.09 (− 0.17, 0)	− **0.17 (**− **0.29, **− 0.04)*	− **0.27 (**− **0.47, **− **0.07)**	− 0.16 (− 0.46, 0.15)
Tolerance : Year (2019)	0 (− 0.19, 0.18)	0.06 (− 0.2, 0.3)	0.07 (− 0.26, 0.4)	0 (− 0.44, 0.42)
Observer Distance (Under 4.5 m) : Tolerance : Year (2019)	**0.25 (0.03, 0.48)**	0.31 (− 0.01, 0.65)	0.49 (− 0.03, 1.02)	0.45 (− 0.31, 1.22)

The first row shows the Bayesian R^2^ values of each model with the estimated error in parentheses. Each cell represents the model estimates with the upper and lower 89% highest density intervals (HDI) in parenthesis, bold cells highlight where HDI parameter values did not include zero. Italic cells highlight when less than or equal to 2.5% of the 89% HDI fell within the − 0.1 to 0.1 range (ROPE) and the probability of direction was greater than 97.5%, which indicates very strong evidence that the most credible parameter values derived from each model are outside of the area equivalent to the null with high certainty of effect existence (i.e., positive/negative direction). Underlined cells highlight where between 2.5% and 10% were within the ROPE and pd was greater than 97.5%. *pd > 97.5% but ROPE > 10%. ^+^pd = 96%, ROPE = 6%.

Figure 1 also highlights that the point where the mean conditional effects for each observer distance (over/under 4.5 m) intersects shifts right along the horizontal axis (towards more tolerant animals) as the proximity buffer is narrowed. For example, for the 5 m and 2.5 m proximity buffers for 2018, the conditional means for over and under 4.5 m intersect near the middle of the tolerance axis, whereas the conditional means for over and under 4.5 m intersect at the highly tolerant end of the axis for the 1 m and touch buffers. Generally, this suggests numerous individuals were observed less frequently as neighbours (i.e., under sampled) when the observer was close versus further away (the mean conditional effect for under 4.5 m was lower than the lower 95% credible interval for when the observer was over 4.5 m for these parts of the tolerance spectrum), and more specifically, that the number of individuals under sampled (when the observer was within 4.5 m) increased as the proximity buffer narrowed.

For 5 m and 2.5 m buffers (across both years) the mean conditional effect for under 4.5 m was higher than the upper 95% credible interval of over 4.5 m at the highest portion (right-side) of the tolerance spectrum. This suggests some individuals may have also been oversampled when the observer was within 4.5 m. In summary, across both years and all proximity buffers, tolerance shared a positive relationship with how often animals occurred as neighbours when the observer was within 4.5 m, but tolerance and occurrences as a neighbour shared a neutral relationship when the observer was further than 4.5 m away.

**Figure 2:**
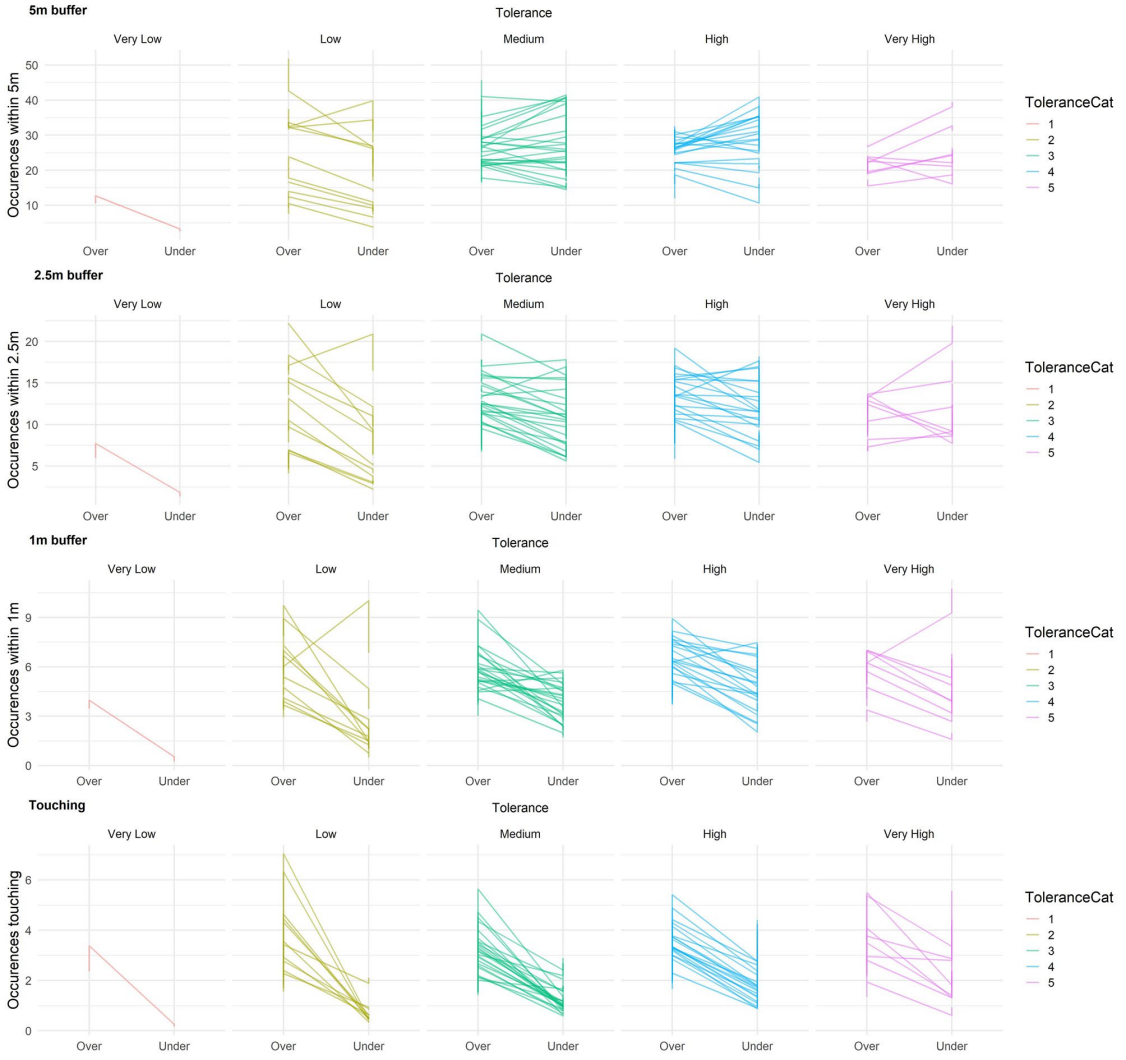
Line graphs representing the predicted individual-level means for occurrences as a neighbour at each observer distance across years. Each row represents the independent proximity buffer models. Within each row tolerance is separated into five equal bins of very low, low, medium, high, and very high based on the tolerance range (− 1.20113 to 0.894895), i.e., the very low category included individuals whose tolerance estimate was between − 1.20113 and − 0.78193. As such, there were an unequal number of individuals within each category.

As population-level parameters are modelled as identical for all individuals, the group-level (or varying) effects of each model also display important information at the individual-level (see Table 2). In general, the among-individual standard deviations were very close to the magnitude of the population-level effects shown in Table 1, suggesting individual identity was an important component governing the number of occurrences as a neighbour. There was no consistent evidence that slopes varied according to year, observer distance, or individual tolerance level (see Table 2: cor estimates), i.e., generally individuals exhibited similar slopes across observation distances and years despite varying tolerance levels. However, specific insights into individual-level effects (see Fig. 2) highlight that several trends underly these results.

**Table 2:** Model summary results for group-level (i.e., individual) effects for all four models, columns represent the data recorded from each proximity buffer.

	5 m Buffer	2.5 m Buffer	1 m Buffer	Touch Buffer
sd (Intercept)	***0.32 (0.25, 0.4)***	***0.37 (0.27, 0.46)***	***0.27 (0.12, 0.44)***	***0.33 (0.14, 0.53)***
sd (Observer Distance)	***0.2 (0.14, 0.27)***	***0.24 (0.15, 0.33)***	0.19 (0, 0.33)*	0.16 (0, 0.31)*
sd (Tolerance)	0.14 (0.04, 0.24)*	**0.23 (0.1, 0.38)**	***0.37 (0.15, 0.59)***	***0.53 (0.23, 0.84)***
sd (Year)	***0.49 (0.27, 0.7)***	***0.53 (0.25, 0.83)***	0.45 (0, 0.8)	0.49 (0, 0.89)*
sd (Observer Distance : Tolerance)	***0.52 (0.29, 0.77)***	**0.54 (0.1, 0.91)**	***1.06 (0.4, 1.7)***	***1.24 (0.23, 2.13)***
cor (Intercept, Observer Distance)	*− ****0.74 (****− ****0.93, ****− ****0.57)***	*− ****0.75 (****− ****0.96, ****− ****0.56)***	− 0.28 (− 0.84, 0.27)	− 0.06 (− 0.72, 0.55)
cor (Intercept, Tolerance)	0.19 (− 0.25, 0.64)	− 0.12 (− 0.53, 0.31)	− 0.22 (− 0.75, 0.26)	− 0.36 (− 0.82, 0.1)
cor (Observer Distance, Tolerance)	− 0.28 (− 0.75, 0.16)	− 0.07 (− 0.58, 0.39)	− 0.25 (− 0.83, 0.28)	− 0.03 (− 0.66, 0.6)
cor (Intercept, Year)	*− ****0.52 (****− ****0.86, ****− ****0.22)***	− **0.43 (**− **0.82, **− **0.05)**	− 0.12 (− 0.68, 0.44)	− 0.15 (− 0.75, 0.4)
cor (Observer Distance, Year)	0.35 (− 0.07, 0.8)	**0.44 (0.02, 0.88)**	− 0.01 (− 0.64, 0.63)	0.01 (− 0.65, 0.63)
cor (Tolerance, Year)	− 0.15 (− 0.72, 0.4)	− 0.08 (− 0.64, 0.49)	0.12 (− 0.46, 0.74)	0.3 (− 0.28, 0.89)
cor (Intercept, Observer Distance : Tolerance)	0.05 (− 0.37, 0.5)	0.09 (− 0.43, 0.64)	0.27 (− 0.25, 0.79)	0.4 (− 0.09, 0.91)
cor (Observer Distance, Observer Distance : Tolerance)	− 0.1 (− 0.53, 0.35)	− 0.12 (− 0.67, 0.4)	0.04 (− 0.53, 0.65)	0.02 (− 0.63, 0.64)
cor (Tolerance, Observer Distance : Tolerance)	− 0.38 (− 0.83, 0.04)	− 0.34 (− 0.84, 0.13)	− **0.46 (**− **0.89, **− **0.08)**	− 0.43 (− 0.91, 0.03)
cor (Year, Observer Distance : Tolerance)	0.15 (− 0.3, 0.63)	0 (− 0.55, 0.54)	0.02 (− 0.55, 0.61)	− 0.3 (− 0.89, 0.25)

Each cell represents the model estimates with the upper and lower 89% highest density intervals (HDI) in parenthesis, bold cells highlight where HDI parameter values did not include zero. Italic cells highlight when less than 2.5% of the 89% HDI fell within the − 0.1 to 0.1 range (ROPE) and the probability of direction was greater than 97.5%. Underlined cells highlight where between 2.5% and 10% were within the ROPE and pd was greater than 97.5%. *pd = 100% but ROPE > 10%.

For the most part, individuals with low and very low observer tolerance seemed to be observed less frequently as neighbours (i.e., under sampled) when the observer was within 4.5 m for all proximity buffers -supporting our initial predictions. For 5 m and 2.5 m buffers, the was a general mix of responses to observer distance across the remaining tolerance categories (see Fig. 2), with examples of some individuals being observed more often (i.e., over sampled) when the observer was within 4.5 m, some individuals being observed similarly across observation distances (i.e., a neutral effect of observation distance), and some individuals being observed less often (i.e., under sampled) when the observer was closer. However, there was a general trend for individuals to occur less frequently when the observer was within 4.5 m as the proximity buffer narrowed. For example, for the 5 m buffer there was generally a neutral or positive effect of the observer being closer on the number of occurrences of ‘medium’ tolerance individuals as neighbours of focal animals, but numerous individuals exhibited negative slopes (i.e., occurred less often when the observer was within versus beyond 4.5 m) for the 2.5 m buffer and very strong negative slopes for the 1 m and touch buffers. Almost all individuals displayed a negative slope for observer distance for the touch models, i.e., they occurred less frequently when the observer was close, suggesting strong evidence that touch associations were under-sampled for the majority of individuals when the observer was within 4.5 m—this is in contrast to our prediction that only intolerant individuals should be affected by observation distances.

### Comparing association matrices collected at different observation distances

We found no evidence of a relationship between proximity association data collected within 4.5 m and beyond 4.5 m of focal animals. This was consistent across both years and all proximity buffers (see Table 3). Although all results were considered statistically significant, this only indicates the robustness of the test statistic and the maximum Mantel test statistic was 0.23 for the touch buffer in 2018, as such, these results indicate that inter-individual association scores varied according to our observation distance, i.e., the association matrices collected from different observation distances were not comparable. This suggests that individuals who occurred frequently as neighbours to specific individuals at one observer distance were not likely to occur at a similar level when observations were undertaken at the alternative distance.

**Table 3:** Mantel test statistics and individual network metrics for each proximity buffer within each year.

Proximity Buffer	Year	Mantel statistic r	Significance	Degree	Closeness	Betweenness
5 m	2018	0.19	0.001	*r*_*s*_ (63) = .18, *p* = .15	r_s_ (63) = .18, p = .15	*r*_*s*_ (63) = .16, *p* = .21
2.5 m	2018	0.16	0.001	*r*_*s*_ (63) = .19, *p* = .14	*r*_*s*_ (63) = .21, *p* = .1	*r*_*s*_ (63) = .22, *p* = .07
1 m	2018	0.15	0.001	*r*_*s*_ (63) = .04, *p* = .73	*r*_*s*_ (63) = .05, *p* < .72	*r*_*s*_ (63) = .08, *p* = .53
Touch	2018	0.23	0.001	*r*_*s*_ (63) = − .06, *p* = .62	*r*_*s*_ (63) = − .13, *p* = .3	*r*_*s*_ (63) = − .07, *p* = .57
5 m	2019	0.16	0.001	*r*_*s*_ (63) = − .01, *p* = .92	*r*_*s*_ (63) = − .0, *p* = .97	*r*_*s*_ (63) = − .01, *p* = .93
2.5 m	2019	0.14	0.001	*r*_*s*_ (63) = − .04, *p* = .74	*r*_*s*_ (63) = − .11, *p* = .38	*r*_*s*_ (63) = − .06, *p* = *.63*
1 m	2019	0.14	0.001	*r*_*s*_ (63) = − .19, *p* = .12	*r*_*s*_ (63) = − .2, *p* = .1	*r*_*s*_ (63) = − .19, *p* = .14
Touch	2019	0.21	0.001	*r*_*s*_ (63) = − .03, *p* = .8	*r*_*s*_ (63) = − .13, *p* = .3	*r*_*s*_ (63) = .0, *p* = .98

Correlations are between data collected within versus beyond 4.5 m. All mantel test statistics were significant (i.e., empirical significance level from 999 permutations was less than 0.01). All individual network metrics were compared using Spearman’s rank tests.

### Investigating whether individuals exhibit similar local positions in networks produced from different observation distances

**Figure 3:**
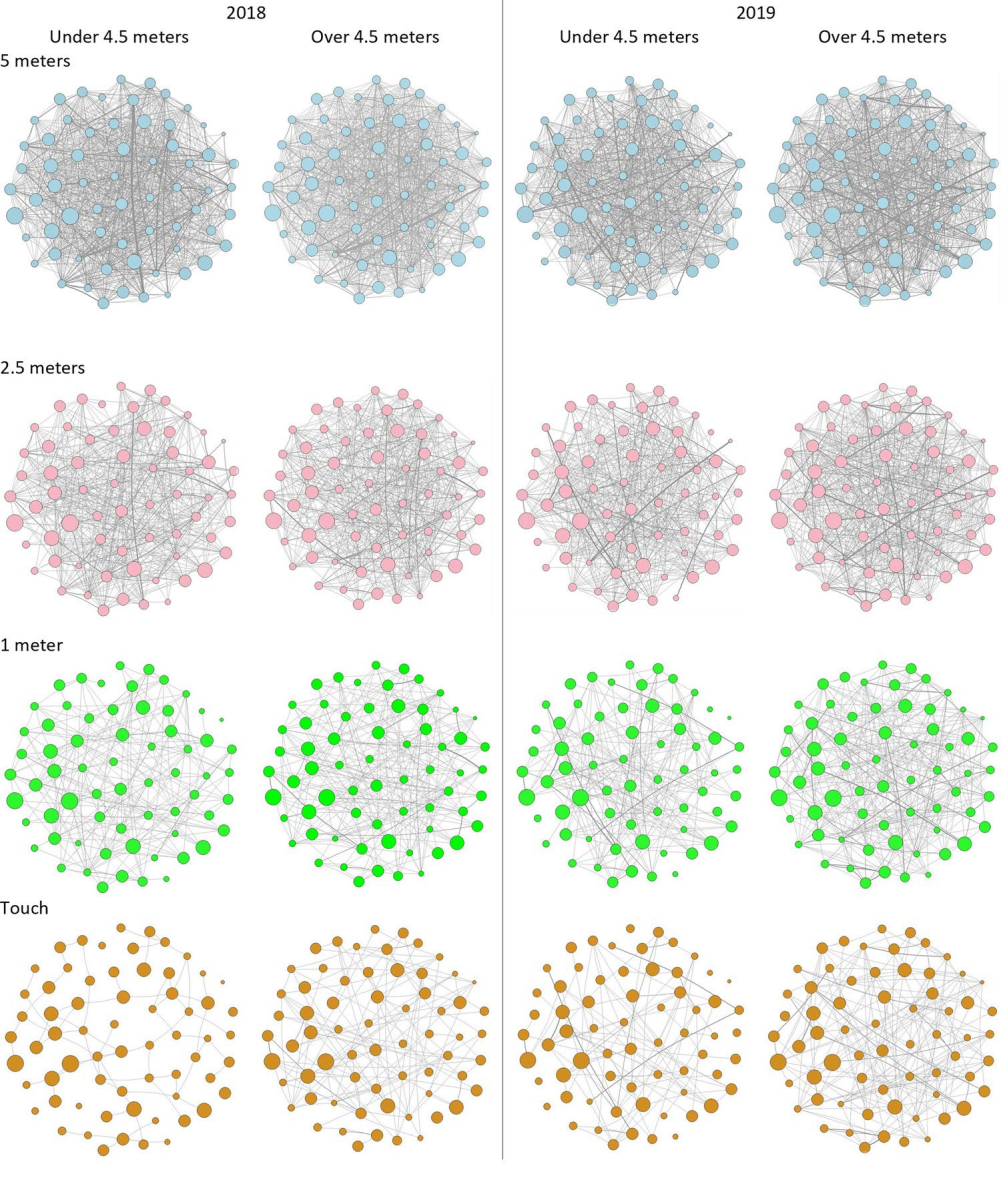
Weighted proximity networks for the habituated baboon group. Rows represent networks based on different proximity buffers. Columns are for each study year and observation distance. Nodes represent individual baboons with their sizes reflecting their individual displacement tolerance estimates (large nodes indicate very high tolerance of observers). Individual node positions were kept constant across every graph to aid visual comparisons. Lines represent proximity associations between the individuals (i.e., a focal animal being observed with the other individual in proximity). The thickness of the lines represents the connection strength between two individuals (i.e., the proportion of each dyad’s focal observations where the two individuals occurred in proximity to one another).

We found no evidence supporting statistically important positive correlations between individual centrality measures. The implication of these results suggests that the number of connections (i.e., degree centrality) each individual has is not consistent across observation distances (see Table 3). In addition, the results for both closeness and betweenness measures suggest individuals are not occupying similar positions in their networks depending on observation distance, and in some cases well connected individuals at one observation distance are poorly connected individuals at the alternative distance, and vice versa (see Fig. 3).

## Discussion

We tested whether the interaction between tolerance and observer distance influenced how often other individuals were observed in proximity to focal animals. At the population-level, there was clear evidence that when the observer was closer (i.e., under 4.5 m) there was a positive association between tolerance and the number of times an individual occurred as a neighbour to focal animals. Group-level results highlighted that a few individuals appeared to be oversampled (relative to the mean) in the 5 m and 2.5 m buffers when the observer was close, including some individuals slightly lower on the tolerance spectrum. Although most individuals exhibited a broadly neutral response to observer distance in the 5 m buffer (i.e., similarly sampled when the observer was within versus beyond 4.5 m), as the proximity buffers narrowed (i.e., 2.5 m, 1 m and, touch) few individuals appeared to remain over or equally sampled, with many animals (not only intolerant individuals) being under sampled. The population-level results highlighted that when the observer was further away (i.e., over 4.5 m) there was generally a neutral relationship between tolerance and occurrences, across both years and all proximity buffers. We also found that association matrices were poorly correlated when data was collected within versus beyond 4.5 m and this was consistent across all proximity buffers and years. Individual network metrics also did not exhibit positive correlations. These results suggest that inter-individual associations would be challenging to sample without bias in this study group unless observation distances were kept above a certain threshold.

Our results suggest that most of our study group were highly habituated, and that ‘low’ relative tolerance (within the study group) may still be indicative of being fairly tolerant of observers. For example, some individuals who had ‘low’ or ‘medium’ tolerance still occurred more frequently in the 5 m buffer when the observer was within 4.5 m. The finding that several animals were oversampled in the 5 m proximity buffer when focal samples were completed at less than 4.5 m may suggest that tolerant phenotypes could be favouring areas near observers. An awareness of tolerance differences could aid subordinate animals in accessing food patches or avoiding aggressions from dominant animals who are less tolerant. Although we found no evidence that dominance rank influenced FID in this study, this does not test whether tolerant animals can exploit a tolerance-derived phenotypic advantage during agonistic or competitive scenarios. This poses an interesting question for future work, in asking whether habituated animals are aware of the tolerance differential between them and use this differential to their advantage.

**Figure 4:**
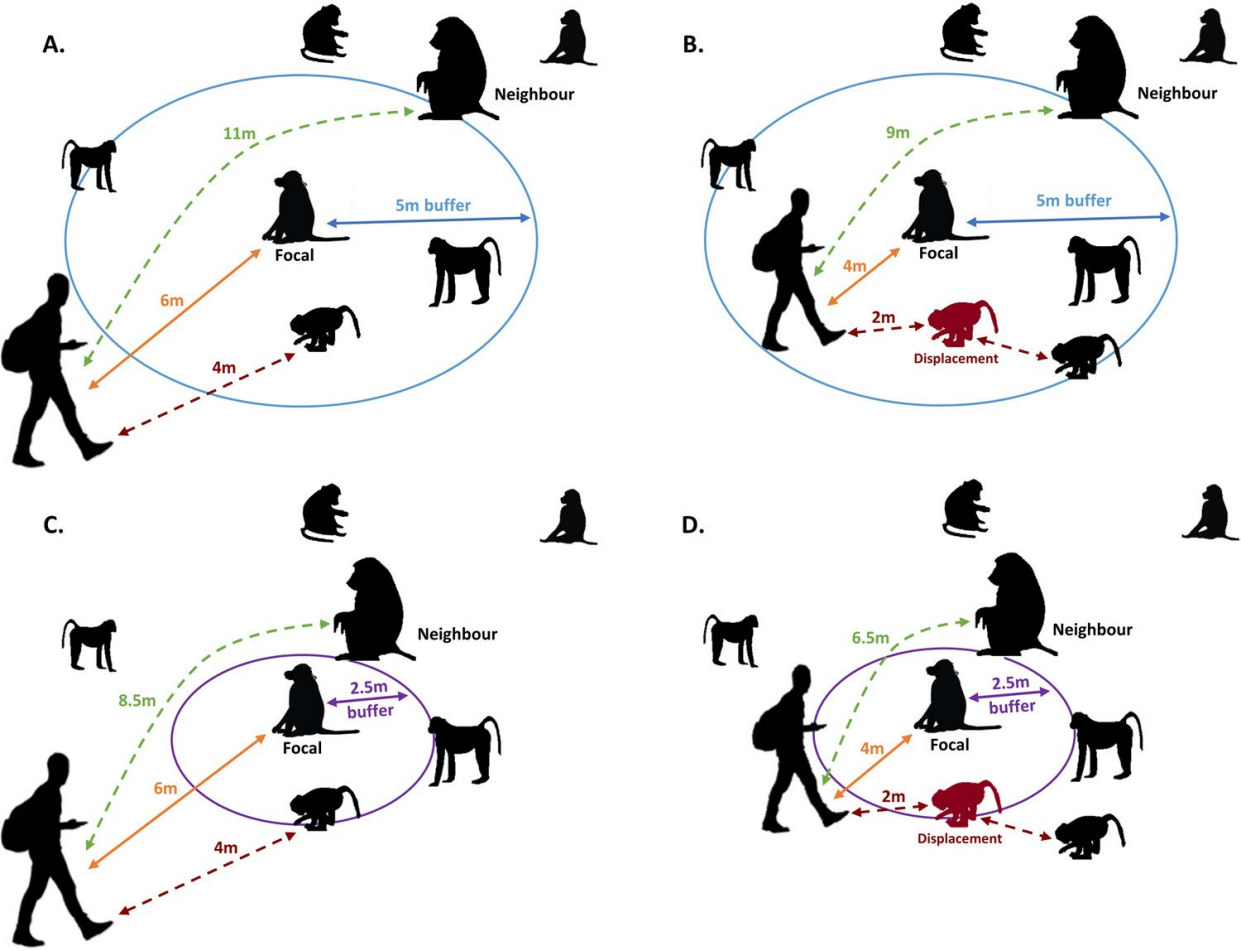
Illustration of the interactive effect of observation distance and proximity buffer. Panel (**A**) highlights that when the observer is 6 m away from the focal animal, a neighbour can still be up to 11 m away from the observer and still be within the 5-m proximity buffer of the focal animal. Narrowing of the observer distance (panel **B**), proximity buffer (panel **C**), or both the proximity buffer and observer distance (panel D) mean that the neighbouring individuals will have to tolerate greater proximity to the observer in order to achieve close proximity to the focal animal. Panels (**B**) and (**D**) also illustrate that when closer observation distances displace neighbouring individuals the likelihood of the displaced individuals still being sampled as neighbours decreases as the proximity buffer narrows, even if the neighbours only slightly adjust their positions.

To our knowledge, this is the first study to demonstrate that highly habituated animals will assort themselves according to tolerance phenotype and observer behaviour. Previous work has highlighted that not all measures of inter-individual association patterns measure the same information, leading to fundamentally different networks^14^; our results suggest observer distance could be just as important methodological information as the parameters used to measure associations. In addition, these results could also explain some of the differences between networks as close proximity or association (e.g., affiliative or grooming interactions) measures may be more susceptible to bias than longer distance rules. Figure 4 illustrates this point and the key findings from our study—although close observer distances can displace individuals regardless of the proximity buffer, the narrower buffers will likely experience greater biases as displacing individuals are more likely to relocate further from the focal animal, thus are less likely to remain within the focal animal’s proximity buffer. Undertaking observations from further away and focusing on larger proximity buffers (e.g., 5 m) may reduce this effect on intolerant animals. Field sites dominated by open habitats may be best placed to do both; undoubtedly, many researchers do this already. However, the specific distances may need testing in each study system and field site, especially where challenging terrain and dense vegetation may limit the practicality of wider proximity buffers and longer observation distances.

The outcome of habituation processes are often classified in qualitative terms, e.g., ‘full habituation’^44^, and such terms have been used even when inter-individual differences in observer tolerance remain (e.g., Ref.^45^). The baboons used in this study received observations for nearly a decade and so had been habituated to researchers over a long period (see Refs.^17,18^). This strongly suggests our group would be considered ‘fully habituated’, and yet consistent inter-individual differences in tolerance to observers remained^2^. Study animals receiving short term or sporadic study attention, or earlier in the habituation process, are unlikely exhibit similarly high tolerance levels; as a result, observer presence and behaviour are likely to have greater impact. In our case, as AA was the main observer over a 4.5-year period (over 400 full-day observations) and completed all focal observations used in this study, inter-observer effects were not considered. However, field sites with regular turn-over in researchers may need to consider whether the interaction between observer distance/behaviour and individual tolerance estimates is consistent across observers as well. Given the complexities of habituation and tolerance, and the variation that likely exists between individuals, groups, populations, and species, it seems important that future research using direct observations on habituated animals routinely measures tolerance levels and communicates the outcomes as vital elements of methodological information going forward.

Collecting data on intolerant animals has always been a challenging task, with some researchers electing to exclude them entirely (e.g., Ref.^46^), however, this strategy does not remove the probability of intolerant animals occurring as neighbours for the remaining (n-1) group members. Excluding individuals based on age classifications (e.g., juveniles) has been shown to create fundamentally different networks^46^; therefore removing individuals based on phenotype will likely introduce a level of bias into networks. Recently, social network research has explored ways of dealing with missing data (see Refs.^47,48^), but when missing data is produced from a non-random process it affects a range of networks and metrics in different ways leading to biased outcomes^49^. In this case, the interaction between tolerance and observer distance appears to be an overlooked ecological driver of inter-individual association patterns in these habituated animals and not a random process. As such, solutions must focus on avoiding sampling bias in situ instead of relying on analytical solutions post-hoc. One option is to explore methods of gathering data without human-presence, with GPS (or radio) collars and camera-trapping being potential solutions^19^. Aside from the ethical concerns associated with collaring^50^, there are challenges with fitting GPS collars to all individuals and numerous reliability issues (e.g., Ref.^51^) which means it is unlikely to offer an immediate solution to the problem of excluding groups of individuals and missing data (e.g., Refs.^46,49^). Similarly, many individuals may be challenging to capture on camera-traps or other automated technologies.

The use of FID methodology has been rare in primatology thus far, and so it is unclear if such methods are viable elsewhere. Future work should only utilise approach methodology if similarly, benign responses are expected across all study subjects initially and throughout all stages of data collection. Tangential approaches and measuring visual orientation/detection (instead of flight/displacement) may be a more viable option if researchers only need to ascertain appropriate observation distances, however, tangential approaches may not elicit responses in very tolerant animals and VOD showed lower within-individual consistencies than FIDs in our previous study^2^. Nevertheless, measuring visual orientation distance using tangential approaches would offer researchers a less demanding method for tracking individual tolerance levels routinely and could be used to understand the differences in habituation and tolerance that may be present across different field sites, species, and individuals through time, although there would need to be consolidation on defining visual orientation for this to be feasible^52^.

Future research should also explore additional methods for investigating habituation, sensitization, and tolerance effects in habituated animals without the use of approach methodology as it is unlikely to be viable in scenarios where animals have become ‘over-habituated’ or aggressive towards humans already. It would be useful to explore and discuss why these tendencies have emerged at certain locations and its impact on the data collected, as it again would suggest researchers are no longer a neutral stimulus (see Ref.^2^). The approach used in this study could also be adapted by having the observer move ahead of the group’s movements to stand in a fixed location as the group passes through. Collecting data on the identities and behaviours (e.g., the frequency or duration of looking bouts ^52,53^) of individuals in proximity to the observer (and the distances between the animals and the observer) could reveal precise information about which individuals tend to adapt their spatial position according to the observer.

Although observer presence may be considered a low level of anthropogenic disturbance, it can have differential impacts on habituated and non-habituated animals^56^. Non-focal animals, particularly predators, are often displaced by human-activities^57^, resulting in increased energetic costs^58^ and potential disruption to vital ecological processes such as predation and competition for resources^19^. Despite our study focusing on habituated animals, we still found that the presence and behaviour (i.e., distance) of observers led to phenotypic assortment. These results demonstrate that overlooking observer factors and tolerance may impact on the quality of data collected, suggesting that future studies need to carefully consider the impacts of observer presence on both focal and non-focal animals.

## Supplementary Information

Supplementary Information 1.

Supplementary Information 2.

## Data Availability

The data required to reproduce the analyses reported in this manuscript are provided as supporting information.
