# Supplementary material for: Intolerant baboons avoid observer proximity, creating biased inter-individual association patterns
Source: Sci Rep. 2022 May 16;12:8077. doi: 10.1038/s41598-022-12312-3 (PMC9110335; doi:10.1038/s41598-022-12312-3)
Supplement: Supplementary file 1 — Supplementary Information 1. [file 41598_2022_12312_MOESM1_ESM.pdf]

Supporting Information

Intolerant baboons avoid observer proximity, creating biased inter-individual association patterns.

Authors

Andrew T. L. Allan[1,2*], Amy F. White[2], Russell A. Hill[1,2,3]

Affiliations

1. Department of Anthropology, Durham University, Dawson Building, South Road, Durham DH1 3LE, UK.

2. Primate and Predator Project, Lajuma Research Centre, PO Box 522, Louis Trichardt 0920, South Africa.

3. Department of Zoology, University of Venda, Private Bag X5050, Thohoyandou 0950, South Africa.

*Corresponding author. Email: andrewtlallan@hotmail.com

Table S1. Summary statistics for the observation distances of each focal individual. Data is split according to the two distinct study periods – 2018 and 2019. Individuals with multiple age-sex classes represent maturation across the categories described in text S1.

		2018					2019				
Focal ID	Age-sex class	Number of focal samples	Min observer distance	Max observer distance	Average observer distance	Standard deviation observer distance	Number of focal samples	Min observer distance	Max observer distance	Average observer distance	Standard deviation observer distance
ARL	J2M	28	2	22	5.59	3.92	25	2	9	4.26	1.74
ATH	AF	28	2.5	18	5.63	3.01	25	2	8.5	4.46	1.68
BAM	J1M	28	1	7	2.54	1.59	25	1	7	3.26	1.73
BIX	J2F/ADF	28	0.5	10	4.61	2.16	25	2	7	4.34	1.64
BOU	AF	28	1	7.5	4.43	1.90	25	1.5	8.5	4.44	2.00
BOX	AF	28	1.5	9	4.30	2.23	25	1	6.5	3.10	1.88
BRA	AF	28	1.5	8	4.25	2.06	25	1	8	3.30	2.07
BRU	AF	28	2	11	4.75	2.13	25	1.5	9	4.32	1.82
BUR	J3M	28	2.5	11.5	5.07	2.10	25	1.5	7	3.78	1.63
CAR	AF	28	1	11	4.63	2.26	25	1	7.5	3.90	2.11
CLO	J2F	28	1	7.5	3.55	1.77	25	1	13	3.36	2.33
COR	AF	28	2	11.5	4.91	2.51	25	1	7	4.40	1.55
DAN	J1F/J2F	28	2	19	4.59	3.25	25	1.5	6.5	3.56	1.57
DAV	AM	28	2	10.5	5.48	1.96	25	2	10.5	4.64	2.03
DIC	J2M/J3M	28	1.5	7	3.80	1.67	25	2	8	4.34	1.57
DIL	J3M	28	3	10	5.30	1.37	25	2.5	7	4.76	1.06
DIN	J2M	28	1.5	15	4.45	2.52	25	1.5	7.5	3.60	1.82
ECH	ADF	28	1	9	4.38	2.34	25	0.5	8.5	3.66	2.08
EGO	AM	28	2.5	9.5	6.21	2.01	25	2	9.5	6.28	1.77
ELA	AF	28	4	11	7.04	1.58	25	4.5	10	7.22	1.48
EVI	ADF/AF	28	1.5	18	4.73	3.23	25	1	7.5	4.26	2.02
FLE	AM	28	1.5	9.5	5.80	1.90	25	2	10.5	4.34	1.98
FUN	J1F/J2F	28	1	12	4.75	2.54	25	0.5	11.5	3.72	2.19
GRO	J1M	28	0.5	16	4.34	2.98	25	0.5	7	3.52	1.92
GRU	AF	28	2.5	11	5.45	2.06	25	1.5	7	3.88	1.38
HEA	AF	28	4	14	7.20	2.59	25	4.5	12	7.24	1.84
HUN	J2M	28	1	8.5	3.29	1.80	25	1	6.5	2.92	1.33
JAC	J2M	28	2	8.5	4.59	1.50	25	1.5	7	3.88	1.76
JOS	AM	28	2	11	6.38	2.52	25	2	8	5.62	1.76
LAR	J1M	28	2	10	4.05	1.67	25	2	6.5	4.38	1.49
LAT	J2M/J3M	28	2	18	4.59	3.00	25	1.5	8	3.78	1.84
LOB	AF	28	2	6.5	3.96	1.31	25	2	8	4.48	1.63
LUK	J3M/ADM	28	1.5	25	5.27	5.14	25	1	7	3.86	1.78
MAN	AF	28	5	10.5	7.29	1.45	25	3.5	15.5	6.30	2.36
MEL	AF	28	4	13.5	7.04	2.53	25	1	8.5	6.08	1.58
MOU	J1M	28	0.5	8.5	3.82	1.87	25	1	6	3.28	1.56
MUR	ADF/AF	28	2.5	10	5.73	1.96	25	1.5	8.5	4.92	2.21
NAT	AM	28	1.5	8.5	5.30	1.84	25	1	7.5	5.10	1.68
NIC	J1M/J2M	28	1.5	12.5	5.04	2.67	25	1	7	4.02	1.52
NOR	AF	28	3.5	11.5	7.59	2.00	25	4	10	6.00	1.60
NOS	AM	28	2.5	12	6.11	2.29	25	2	8.5	4.82	1.93
PIX	AF	28	2	14	5.05	3.17	25	2	10	4.66	2.09
PON	J1M/J2M	28	0.5	8.5	3.86	1.86	25	1	7	3.50	1.83
PRA	J2M/J3M	28	1.5	11	4.25	2.15	25	1.5	6.5	3.96	1.53
PRE	ADM/AM	28	1.5	14	6.38	2.84	25	2	10	5.56	1.86
RHO	ADF/AF	28	1.5	12	3.61	2.18	25	1.5	7	3.86	1.64
SAC	J2F	28	1.5	9.5	5.02	1.88	25	2	9	4.54	1.90
SAN	J2F/ADF	28	1	11	4.68	2.22	25	1	6.5	3.38	1.63
SCA	AF	28	1	8	3.75	1.98	25	1	6.5	3.58	1.55
SCO	J2M	28	1	11	4.20	2.55	25	1.5	9	4.00	1.82
SEX	AM	28	2.5	10	6.05	1.82	25	2.5	7	5.22	1.32
SIL	AF	28	2.5	11	5.63	1.77	25	1	7	4.24	1.70
SLI	AF	28	2.5	7.5	4.63	1.53	25	1.5	9	4.70	2.12
STE	J2M	28	1	10	2.96	1.93	25	1	6	3.14	1.67
STI	J1F/J2F	28	0.5	8	3.63	2.14	25	1	5.5	2.94	1.26
STR	J3M	28	1.5	9.5	4.63	2.18	25	1	8	4.42	1.80
TER	AF	28	4	20	8.77	3.67	25	5	9.5	7.00	1.20
THI	AF	28	2	11	4.95	2.23	25	1	9	4.64	2.14
TIL	J2F/ADF	28	2	8	4.29	1.81	25	0.5	7	2.94	1.76
TON	J1F	28	1	13	4.45	2.37	25	1	12	3.98	2.63
TRI	AF	28	0.5	6.5	3.84	1.53	25	1	7.5	3.68	1.96
TRU	J1F	28	1.5	8.5	4.09	1.88	25	1	7	3.10	1.56
TUP	AF	28	1.5	8	4.80	1.69	25	1	8	4.30	1.82
YOD	J1F/J2F	28	2.5	10.5	5.66	2.37	25	2	18	5.70	3.39
YOL	AF	28	4.5	18	10.18	3.42	25	4.5	15	8.98	2.43

Text S1. Descriptions of the various habitat types categorised in this study

Habitat type – the Lajuma field site and surrounding properties contain a range of habitat types that can be broadly classified into several categories: forest, woodland, bushland, camp, farm, marshland, grassland, rock and cliffs. These habitat types will vary in their respective structure and therefore influence visibility to a different extent.

“Closed” habitats

Forest: An area composed largely of trees with overlapping crowns forming 60-100% cover. Trees will be mostly tall providing extensive and near continuous shade.

Woodland: Canopy is more open than forest, with 25-60% cover, allowing sunlight to penetrate between the trees. Woodlands may support an understory of shrubs, herbs, or grasses.

Bushland: An area where shrubs or are the dominant vegetation. A shrub is a woody perennial plant, smaller than a tree, with several major branches arising near the base of the stem. Areas of extensive tree regeneration, i.e., saplings, can also qualify as bush.

“Open” habitats

Grassland or savannah: Open area covered predominantly with grass. These areas may be devoid of trees entirely but can also contain widely spaced trees with a minimum of 5% cover to a maximum of 25% cover.

Marshland: Exclusively found in flat regions along permanent water streams on peat. Vegetation components of marshlands include reeds, sedges, and grasses. Marshland was combined with grassland for table S2.

Rocks: Areas where ground predominantly consists of rocks and boulders, rather than soil.

Road: Dirt roads that run through the study area. Very little traffic (less than 5 vehicles a day) and always at low speeds (less than 10mph). Road use often offers a localised enhancement in visibility for the baboons.

Camp: Used or disused human settlements on Lajuma and neighbouring properties.

Farm: Ottosdal Macadamia farm or area around Ottosdal farmhouse.

Text S2. Summary of focal observation distances by habitat type and scan data exploring spatial cohesion (i.e., inter-individual proximity patterns) of the group by habitat type.

Previous study

Between 2015 and 2018 we used instantaneous scan sampling to collect behavioural data on the baboons at 20-minute intervals throughout the day (e.g., 06:00, 06:20, 06:40 etc). We recorded the behaviour, habitat use, and number of neighbours (within 5 meters) of as many individuals as possible within 10-minute sampling periods. We found that the spatial cohesion patterns (i.e., inter-individual spacings) were generally similar across the range of habitats the baboons utilised, see table S2 – average and standard deviation number of neighbours within 5 meters for 2015 – 2018 scan sampling.

Current study

Given our previous findings we therefore did not attempt to sample evenly across the habitat types. Despite this, we still found that average observation distance was consistent across habitat types and years, as was the distribution of focal samples within versus beyond 4.5-meter observation distances for each habitat type (see table S2).

Table S2. Summary of spatial cohesion patterns from the previous study (2015 – 2018 scan sampling) and observation distances for the current study (2018 and 2019 focal sampling). For the 2015 – 2018 study the average (and standard deviation) number of neighbours within 5 meters was calculated from 110,997 individual instantaneous scan samples. For the 2018 and 2019 focal sampling, number within and beyond 4.5m refer to the total number of focal samples completed with the observer within versus beyond 4.5 meters.

	2015 - 2018 scan sampling		2018 focal sampling				2019 focal sampling			
	Average number of neighbours within 5m	Standard deviation number of neighbours within 5m	Average observer distance	Standard deviation observer distance	Number within 4.5m	Number beyond 4.5m	Average observer distance	Standard deviation observer distance	Number within 4.5m	Number beyond 4.5m
Bushland	2.07	1.96	4.85	2.88	49	39	4.02	1.96	38	24
Camp	2.18	2.09	5.55	2.12	45	74	4.8	2.02	60	80
Farm	1.62	1.78	6.42	3.99	12	25	5.48	2.18	20	49
Forest	2.19	2.25	5.22	2.46	36	50	4.63	1.99	67	85
Grassland	1.81	1.93	5.08	2.63	362	370	4.13	2.04	326	265
Road	2.06	2.22	5.03	2.85	74	60	4.68	2.64	61	73
Rock	2.77	2.66	6.1	3.51	33	54	5.17	2.34	51	84
Woodland	1.89	1.84	4.58	2.34	307	230	4.3	3.58	186	156

Text S3. Age-sex class categories and descriptions

Female baboons:

AF (Adult female) – Attainment of full body size, either cycling regularly, pregnant or lactating. Nipples also enlarge and elongated from suckling infants.

AFI (Adult female with infant) – As above but with their own neonate or dependent infant. Approaches were not completed on individuals known to have dependent infants but when those infants were not attached (e.g., suckling, being carried dorsally or ventrally) to the mother.

ADF (Adolescent Female) – Nearly adult female size, with the onset of the first sexual swellings. If visible, nipples are much smaller and button-like than that of an adult female.

Male baboons:

AM (Adult male) – All secondary sexual characteristics fully grown, musculature (most noticeably in chest and rump) expands to full adult size.

ADM (Adolescent Male) – Massive growth in secondary sexual characteristics; testes expand, canines and mane grow longer, body size increases to near that of an adult male.

J3M (Juvenile 3 Males Only) – Body size that of an adult female, muzzle further extended to nearly that of an adult male. Testes start to expand and are clearly visible. Mane becomes noticeable.

Juvenile baboons of both sexes:

J2M/F (Juvenile 2) – Little demarcation from previous period, with greater body size. Hair becomes darker, changing to a more adult grey/brown colouration.

J1M/F (Juvenile 1) – Little demarcation from infants, but fully weaned and nutritionally independent. Muzzle starts becoming more elongated and pronounced. Pelage is still lighter than in juvenile 2. Male/female distinction based on genitalia and noticeable absence/presence of a separation in the callosities.

Text S4. Updating the FID model to include dominance rank, age-sex class, and individual trial number per observation day

Methods

The model used to calculate displacement tolerance estimates (i.e., conditional modes) was updated from our previous study (i.e. Ref.[1]) to include population-level (i.e., fixed) effects for age-sex class (see text S1 for descriptions) and dominance rank as both could be important phenotypic factors to consider in behaviours that may have personality components [2,3]. Rank was calculated using the *isi13* function from the *compete* package[4], based on all displacement, supplant, and agonistic dyadic events between April 2017 and April 2018 (n=908); fights between males were excluded as the 'winner' can often be subjective during conflict. We additionally included an additive population-level effect for individual trial number per day to ensure habituation and sensitization effects were explored effectively across all temporal levels.

As with our previous study, we also included the population-level effects of engaged/not engaged (behaviour), habitat (open/closed), height (ground/above ground), number of neighbours within 5 meters, neighbour flee first, and external events within 5 minutes. Date was also included as a random effect crossed with individual identity. Visual orientation distance index (difference between the distance at which the focal animal visually oriented towards the approaching observer and FID) was included as a population-level effect and random slope over individual identity to control for issues relating to the envelope constraint[5,6]. Although we previously found no evidence of habituation or sensitization effects within individuals or across the group, we also retained these factors to ensure habituation and sensitization effects were explored effectively across all temporal levels. The updated model therefore also included an interaction between observer identity and individual trial number per observer as both population-level effects and random slopes over individual identity. In all cases, random slopes were modelled with correlated intercepts to ensure the estimated model captured all levels of by-individual variation.

Statistical analysis

The updated model was fit using the *brm* function from the *brms* package[7] in the software R [8]. Each model was run for six Hamiltonian Markov chains for 15000 iterations, with warmup iterations set to 5000 and adapt_delta to 0.95. All these parameters were set higher than default to allow algorithms to converge efficiently, producing robust posterior samples[7,9]. The model was fit with a Log-normal response distribution and default link function. Normal priors (mean = 0, standard deviation = 100) were assigned to all population-level effects, whilst the remaining model components were assigned default Student t priors (df = 3, mean = 0, scaling factor = 10), however, in the case of the standard deviations of group-level (i.e., random) effects these parameters are constrained to be positive and therefore a half Student-t prior is implemented. None of the population-level effects were mean-centred as they were either count or categorical variables, with the exception of visual orientation distance index (VODI) which we kept in raw format to ensure it was on the same scale as the response variable to effectively control for the envelope constraint[1,6]. All model fits were assessed as described in the main text using graphical posterior predictive checking (pp_check), examining trace plots, Rhat values, and the bulk and tail effective sample sizes. We also assessed for multicollinearity issues.

As this updated model was very complex it was necessary to compare its predictive precision to the previous model to ensure we selected a model that performed well for conditional modes estimation. We therefore estimated the pointwise out-of-sample prediction accuracy from each

model using leave-one-out cross-validation (LOO) from the ‘loo’ package [10]. ‘loo’ uses a Pareto smoothed importance sampling (PSIS) procedure for regularising importance weights when computing LOO [11]. PSIS approximation reliability was confirmed by inspecting the estimated shape parameter $\hat{k}$ diagnostic values in the generalized Pareto distribution [11,12]. The LOO process uses n-1 sample points (focal observations) to tune a specific algorithm to predict the left-out point, allowing the n-1 samples to act as a training set for optimising the free parameters of the model and assess how well the tuned algorithm performs at predicting the left-out sample point. This process is repeated for the remaining samples and produces a test performance for all samples within each model, the resultant estimates therefore provide ordinal information about relative model prediction performance.

Bayesian stacking was undertaken using the ‘loo_model_weights’ function from the “loo” package. When comparing two (or more) models using stacking with PSIS-LOO values, stacking utilises the data produced from the PSIS-LOO procedures of each candidate model and compares the performance and accuracy of each model at predicting each left out sampling point. To achieve a stacking weight of 1, a model needs to predict every left-out data point with greater accuracy than the remaining models in the stack. Thus, models with greater weight offer greater predictive performance over other models exhibiting lower weight [12].

Results

The stacking weights of our original model (i.e., from [1]) was 0.692, whilst the updated model (including age-sex class, dominance rank, and individual trial number per day) received a weight of 0.308, with both producing identical Bayesian R^2 values (see main text for explanation) of 0.67 (0.021) and 0.67 (0.022) for the original and updated models respectively. See tables S3 and S4 for model summary results. Given the similar predictive performance of both models we elected to use the updated model (table S4) as it included additional factors that could account for methodological (trial number per individual per day) and phenotypic sources of variance (age-sex class and dominance rank), the latter of which are important in studies where behaviours may have personality components.

Table S3. FID model summary for model specification used in [1]. Parameter estimates for the model describing the relationship between FID and the predictor variables.

**Population-level effects**	Estimate	Est.Error	L-95% CI	U-95% CI	Rhat	Bulk_ESS	Tail_ESS
Intercept	0.67	0.1	0.47	0.87	1.00	10517	22924
VODI	-0.04	0.01	-0.07	-0.01	1.00	41110	45536
Not engaged (Behaviour)	0.14	0.02	0.1	0.18	1.00	78223	46542
Open (Habitat)	0.12	0.02	0.08	0.16	1.00	79882	48533
Ground (Height)	0.12	0.06	0	0.23	1.00	83916	48204
Number of neighours	-0.08	0.01	-0.09	-0.06	1.00	82712	47203
Neighbour flee first	0	0.05	-0.1	0.09	1.00	88317	45595
External events within 5 minutes	0.01	0.04	-0.06	0.08	1.00	86039	46842
Unfamiliar observer (AB)	-0.14	0.09	-0.3	0.04	1.00	16660	26274
Individual trial number per observer	-0.02	0.01	-0.04	-0.01	1.00	17167	27184
Unfamiliar observer (AB) : Trial number per observer	0.02	0.01	0	0.05	1.00	16108	26709
**Family specific (log-normal)**							

Sigma	0.36	0.01	0.34	0.37	1.00	52018	44512

Group-level effects

Date (58 levels)							
sd(Intercept)	0.14	0.02	0.11	0.19	1.00	16885	31258
Individual identity (69 levels)							
sd(Intercept)	0.49	0.05	0.4	0.6	1.00	11274	23982
sd(VODI)	0.06	0.02	0.02	0.1	1.00	10813	15497
sd(Observer ID (Unfamiliar))	0.18	0.04	0.1	0.26	1.00	12110	11018
sd(TrialNo)	0.01	0	0	0.02	1.00	9285	10989
sd(Observer ID (Unfamiliar) : TrialNo)	0.01	0.01	0	0.02	1.00	7293	13543
cor(Intercept,VODI)	0.26	0.22	-0.15	0.69	1.00	19634	24009
cor(Intercept, Observer ID (Unfamiliar))	0.04	0.2	-0.34	0.44	1.00	28093	30814
cor(VODI,ObserverAB)	0.16	0.28	-0.38	0.68	1.00	9008	15950
cor(Intercept,TrialNo)	-0.46	0.25	-0.84	0.15	1.00	37460	27306
cor(VODI,TrialNo)	-0.25	0.33	-0.8	0.44	1.00	15557	27136
cor(Observer ID (Unfamiliar),TrialNo)	-0.39	0.29	-0.86	0.27	1.00	19106	28657
cor(Intercept, Observer ID (Unfamiliar) : TrialNo)	-0.12	0.33	-0.73	0.55	1.00	36243	36483
cor(VODI, Observer ID (Unfamiliar) : TrialNo)	-0.36	0.35	-0.88	0.47	1.00	23614	33416
cor(Observer ID (Unfamiliar), Observer ID (Unfamiliar) : TrialNo)	-0.05	0.37	-0.69	0.69	1.00	26774	36387
cor(TrialNo, Observer ID (Unfamiliar) : TrialNo)	-0.13	0.39	-0.78	0.67	1.00	14889	30011

Table S4. Updated FID model summary. Parameter estimates for the model describing the relationship between FID and the predictor variables. This model included additional population-level effects for dominance rank, age-sex class, and individual trial number per observation day that were not included in the previous study[1].

Population-level effects

	Estimate	Est.Error	L-95% CI	U-95% CI	Rhat	Bulk_ESS	Tail_ESS
Intercept	0.59	0.22	0.16	1.02	1.00	11150	20219
VODI	-0.04	0.01	-0.07	-0.01	1.00	45089	43843
Not engaged (Behaviour)	0.14	0.02	0.1	0.18	1.00	75676	46742
Open (Habitat)	0.12	0.02	0.08	0.17	1.00	74456	47230
Ground (Height)	0.1	0.06	-0.01	0.22	1.00	79393	47027
Number of neighours	-0.08	0.01	-0.09	-0.06	1.00	79612	47833
Neighbour flee first (Yes)	0	0.05	-0.09	0.09	1.00	79026	47247
External event within 5 minutes (Yes)	0.01	0.04	-0.06	0.08	1.00	78334	45560
Observer identity (Unfamiliar)	-0.13	0.09	-0.3	0.04	1.00	17579	26863
Individual trial number per observer	-0.02	0.01	-0.04	-0.01	1.00	19583	29511
Individual trial number per day	-0.04	0.03	-0.1	0.02	1.00	76460	47058
Dominance rank	0	0	-0.01	0	1.00	13204	22539
Adolsecent males	0.12	0.23	-0.33	0.57	1.00	11360	19989
Adolsecent females	0.37	0.14	0.08	0.65	1.00	12449	22515
Adult females with infants	0.43	0.14	0.14	0.7	1.00	13101	23639
Adult males	0.48	0.23	0.01	0.93	1.00	10857	19694
Juvenile females (J1F)	0.2	0.21	-0.21	0.61	1.00	11848	23020

Juvenile males (J1M)	-0.03	0.19	-0.4	0.33	1.00	9583	17101
Juvenile females (J2F)	0.04	0.21	-0.37	0.45	1.00	11662	22725
Juvenile males (J2M)	0.14	0.19	-0.24	0.5	1.00	9515	18223
Juvenile males (J3M)	0.23	0.2	-0.18	0.62	1.00	10188	18948
Observer ID (Unfamiliar) : Trial number per observer	0.02	0.01	0	0.05	1.00	16586	26009
**Family specific (log-normal)**							
Sigma	0.36	0.01	0.34	0.37	1.00	45504	44918
**Group-level effects**							
Date (58 levels)							
sd(Intercept)	0.14	0.02	0.11	0.19	1.00	16119	29629
Individual identity (69 levels)							
sd(Intercept)	0.44	0.05	0.35	0.55	1.00	17162	27204
sd(VODI)	0.06	0.02	0.02	0.09	1.00	13279	14453
sd(Observer ID (Unfamiliar))	0.16	0.04	0.07	0.24	1.00	14552	12602
sd(TrialNo)	0.01	0	0	0.02	1.00	11312	19684
sd(Observer ID (Unfamiliar) : TrialNo)	0.01	0.01	0	0.02	1.00	8721	15441
cor(Intercept,VODI)	0.24	0.23	-0.21	0.68	1.00	23434	30400
cor(Intercept, Observer ID (Unfamiliar))	0.2	0.22	-0.23	0.64	1.00	23188	29020
cor(VODI, Observer ID (Unfamiliar))	0.15	0.3	-0.44	0.7	1.00	12181	23006
cor(Intercept,TrialNo)	-0.38	0.32	-0.85	0.4	1.00	33751	32637
cor(VODI,TrialNo)	-0.13	0.37	-0.78	0.6	1.00	26874	35806
cor(Observer ID (Unfamiliar),TrialNo)	-0.28	0.34	-0.84	0.47	1.00	27650	38149
cor(Intercept, Observer ID (Unfamiliar) :TrialNo)	-0.17	0.31	-0.73	0.47	1.00	30434	34016
cor(VODI, Observer ID (Unfamiliar) :TrialNo)	-0.4	0.33	-0.89	0.38	1.00	21271	29513
cor(Observer ID (Unfamiliar), Observer ID (Unfamiliar) :TrialNo)	-0.05	0.36	-0.66	0.69	1.00	28788	37424
cor(TrialNo, Observer ID (Unfamiliar) :TrialNo)	-0.14	0.39	-0.79	0.67	1.00	13992	31583

We found no evidence that the study subjects habituated or sensitized across any timeframe to the approaches completed previously (see table S3 and S4). Ongoing monitoring of study subjects' behavioural responses also validated this[1], providing strong evidence that the methods did not create stress or anxiety in the study subjects. Adult females with and without infants, and adult males generally exhibited slightly longer FIDs than younger individuals (see table S4), although the credible intervals overlapped with other age-sex classes. The other age-sex classes produced generally similar FIDs with wide credible intervals overlapping with one another (see fig S1). However, there was a general trend for FIDs to increase with age-sex class category in male individuals (i.e., mean conditional effect of FID in adult males > juvenile-3 males > J2M > J1M). This was not the case in female individuals as the mean conditional effect of FID was higher for all juvenile females than adolescent females. We found no evidence that dominance rank (estimate 0 lower and upper credible intervals: -0.01, 0) or individual trial number per day (-0.04, -0.09, 0.02) were important predictors of FID.

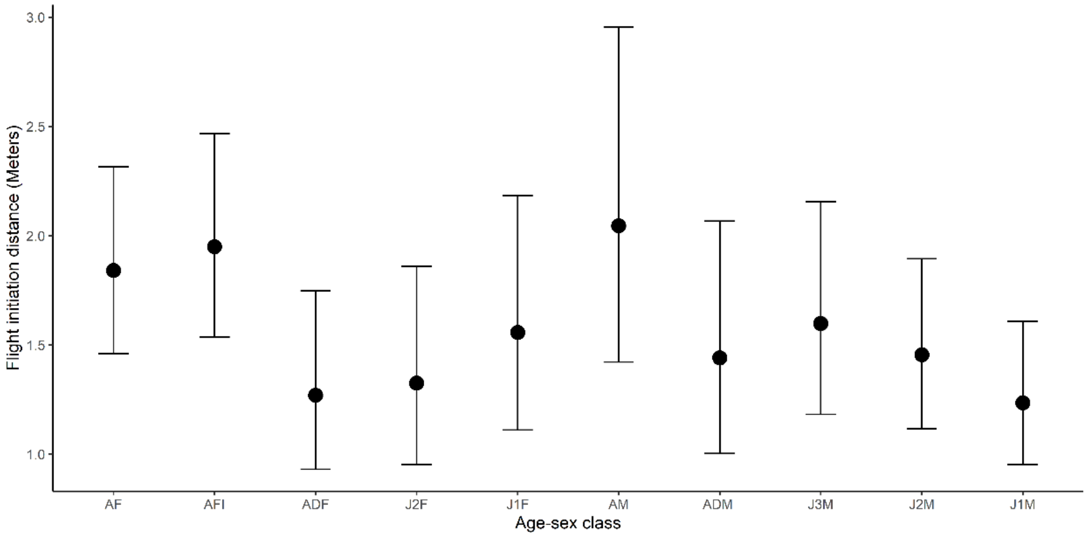

Fig S1. Conditional effects plot for each age-sex class and flight initiation distance. The mean was used as the measure of central tendency, 2.5 and 97.5% percent credible intervals also displayed. From left to right on the x-axis: adult females without infants (AF), adult females with infant (AFI), adolescent females (ADF), juvenile-2 females (J2F), juvenile-1 females (J1F), adult males (AM), adolescent males (ADM), juvenile-3 males (J3M), juvenile-2 males (J2M), and juvenile-1 males (J1M) (see text S2 for descriptions).

Table S5. Summary statistics and conditional modes for individual baboons. Conditional modes are derived from the original FID model[1] and from the updated model used in this study which included population-level additive effects for age-sex class, rank, and individual trial number per day (see text S3 and table S4). Numbers in parentheses refer to the standard deviation associated with the conditional modes (mean individual differences to the population-level mean). *Highlights individuals who disappeared after FID trials were completed and were not included in the occurrences study.

ID	Age-sex class	Number of approaches	Min start distance	Max start distance	Average start distance	Minimum FID	Maximum FID	Mean FID	Median FID	Standard deviation FID	Conditional modes (from [1])	Conditional modes (Updated model)
ARL	J2M	24	2.532	9.587	5.103	0.974	5.011	2.064	1.808	0.875	-0.042 (0.12)	-0.084 (0.15)
ATH	AF	24	3.076	13.161	6.038	0.809	7.429	3.205	3.031	1.568	-0.48 (0.13)	-0.29 (0.16)
BAM	J1M	24	2.941	9.573	4.749	0.304	1.933	0.934	0.843	0.438	0.841 (0.13)	0.591 (0.16)
BIX	J2F	24	3.112	9.494	5.471	1.217	3.99	2.505	2.564	0.744	-0.259 (0.12)	-0.403 (0.18)
BLO*	AM	24	3.483	9.261	5.351	1.657	5.965	2.645	2.369	1.05	-0.226 (0.12)	0.11 (0.17)
BOU	AF	24	2.837	10.323	5.105	0.564	1.81	1.104	1.038	0.34	0.557 (0.13)	0.614 (0.16)
BOX	AF	24	2.607	9.127	4.973	0.514	1.89	1.064	0.987	0.398	0.525 (0.13)	0.62 (0.16)
BRA	AF	24	3.114	10.677	5.271	0.475	1.849	1.152	1.126	0.355	0.516 (0.13)	0.331 (0.18)
BRU	AF	24	3.115	9.868	5.22	0.38	2.255	1.264	1.311	0.531	0.305 (0.15)	0.447 (0.16)
BUR	J2M	24	3.305	9.687	5.652	0.935	4.762	2.356	2.039	0.971	-0.142 (0.12)	-0.157 (0.16)
CAR	ADF	24	3.289	9.843	5.312	0.4	2.482	1.13	1.09	0.491	0.649 (0.13)	0.441 (0.18)
CLO	J1F	24	2.919	8.42	5.165	0.493	3.232	1.442	1.31	0.714	0.448 (0.13)	0.274 (0.16)
COR	ADF	24	3.232	9.333	5.639	0.976	4.023	2.076	1.938	0.872	-0.082 (0.13)	-0.248 (0.18)
CRO*	ADM	24	3.31	7.571	5.088	1.105	4.753	2.343	2.185	0.88	-0.196 (0.12)	-0.196 (0.18)
DAN	J1F	24	3.087	11.842	5.668	0.759	4.046	1.989	1.825	0.939	-0.046 (0.13)	-0.014 (0.21)
DAV	AM	24	3.412	9.922	5.25	0.848	4.296	1.715	1.618	0.716	0.106 (0.13)	0.421 (0.18)
DIC	J1M	24	2.779	9.932	5.411	1.016	5.773	2.498	2.341	1.132	-0.052 (0.12)	-0.33 (0.17)
DIL	J2M	24	2.91	8.466	5.891	1.428	5.007	2.76	2.41	1.086	-0.229 (0.12)	-0.187 (0.15)
DIN	J1M	24	2.817	10.35	5.503	0.622	4.334	1.983	1.68	1.054	0.164 (0.13)	-0.056 (0.16)
ECH	ADF	24	2.907	8.948	4.696	1.126	4.031	1.9	1.837	0.585	0.057 (0.12)	-0.178 (0.18)
EGO	AM	24	3.329	11.272	6.162	1.678	7.148	3.514	3.457	1.306	-0.581 (0.13)	-0.226 (0.17)
ELA	AF	24	3.433	13.646	7.909	2.469	10.254	5.334	4.445	2.064	-0.902 (0.13)	-0.671 (0.16)
EVI	AF	24	3.001	10.586	5.883	0.661	4.798	1.701	1.333	1.106	0.335 (0.13)	0.099 (0.18)
FLE	AM	24	3.609	10.26	5.675	1.423	5.748	2.674	2.645	0.896	-0.308 (0.13)	-0.005 (0.17)
FUN	J1F	24	2.889	9.993	5.141	0.43	3.004	1.253	1.044	0.663	0.605 (0.13)	0.558 (0.18)
GRO	J1M	24	3.026	10.889	5.192	1.058	3.343	1.926	1.791	0.663	0.037 (0.13)	-0.247 (0.17)
GRU	AF	24	2.937	10.995	5.476	0.935	3.126	1.982	1.992	0.562	-0.071 (0.12)	0.017 (0.16)
HEA	AF	24	3.14	12.728	6.694	1.473	9.544	3.983	3.486	1.957	-0.618 (0.13)	-0.463 (0.15)
HEN*	ADM	24	2.739	10.771	5.442	1.203	5.341	2.371	2.264	0.921	-0.104 (0.12)	-0.069 (0.15)
HUN	J2M	24	3.001	8.138	4.97	0.365	2.36	1.353	1.288	0.533	0.375 (0.13)	0.275 (0.17)
JAC	J2M	24	3.075	14.242	5.819	0.958	6.224	2.64	2.299	1.16	-0.2 (0.12)	-0.291 (0.17)
JOS	AM	24	3.37	10.806	6.316	1.267	6.4	2.997	2.681	1.133	-0.307 (0.12)	0.019 (0.17)
LAR	J1M	24	3.17	10.861	5.632	1.016	7.663	2.917	2.244	1.81	-0.199 (0.13)	-0.43 (0.15)
LAT	J1M	24	3.342	9.447	5.082	0.535	4.236	1.901	1.814	0.802	-0.038 (0.13)	-0.234 (0.15)
LOB	AF	24	2.748	8.693	5.001	0.862	2.675	1.529	1.466	0.409	0.192 (0.12)	0.327 (0.14)
LUK	J3M	24	2.73	9.272	5.223	0.722	2.417	1.654	1.599	0.47	0.204 (0.12)	0.226 (0.15)
MAN	AF	24	3.504	13.452	6.637	2.002	8.418	4.11	3.794	1.733	-0.74 (0.13)	-0.494 (0.16)
MEL	AF	24	3.239	12.28	7.024	1.321	9.099	3.64	3.318	1.642	-0.611 (0.13)	-0.403 (0.14)
MOU	J1M	24	3.007	8.644	4.869	0.462	2.432	1.103	1.059	0.378	0.61 (0.12)	0.368 (0.15)
MUR	ADF	24	3.392	10.478	5.444	1.467	7.426	2.555	2.264	1.256	-0.203 (0.12)	-0.433 (0.18)
NAT	ADM	24	3.507	11.524	5.668	0.728	2.55	1.513	1.507	0.456	0.226 (0.12)	0.196 (0.18)
NIC	J1M	24	3.407	11.479	5.933	0.951	8.566	2.921	2.478	1.494	-0.295 (0.12)	-0.464 (0.16)
NOR	AF	24	4.132	12.279	7.237	2.165	7.503	4.525	4.369	1.377	-0.822 (0.13)	-0.581 (0.18)
NOS	AM	24	3.17	17.082	6.589	1.156	7.073	2.859	2.59	1.314	-0.126 (0.13)	0.201 (0.18)
PIX	AF	24	3.265	9.964	5.853	1.18	4.333	2.105	1.841	0.817	-0.022 (0.13)	0.162 (0.15)
PON	J1M	24	2.747	9.63	4.897	0.369	2.874	1.044	0.855	0.569	0.815 (0.14)	0.566 (0.17)
PRA	J3M	24	2.69	8.85	5.18	0.492	2.734	1.752	1.795	0.552	0.09 (0.13)	0.101 (0.16)
PRE	ADM	24	2.98	10.436	5.432	0.805	3.505	2.286	2.443	0.702	-0.159 (0.13)	-0.077 (0.17)
RHO	ADF	24	2.762	8.535	4.909	0.749	2.328	1.538	1.552	0.441	0.236 (0.12)	-0.015 (0.18)
RIP*	AF	24	3.359	10.115	6.453	1.267	6.449	3.091	2.87	1.471	-0.3 (0.13)	-0.16 (0.15)
SAC	J2F	24	2.922	8.404	5.194	0.997	3.659	1.916	1.769	0.764	0.02 (0.13)	-0.2 (0.17)
SAN	J2F	24	2.751	10.055	5.357	0.808	5.258	2.24	1.878	1.221	-0.011 (0.13)	-0.199 (0.18)
SCA	AF	24	2.946	9.306	5.075	0.217	1.854	0.912	0.722	0.494	0.798 (0.14)	0.895 (0.15)
SCO	J1M	24	2.769	9.309	5.04	0.28	3.678	1.188	1.05	0.648	0.54 (0.13)	0.273 (0.17)
SEX	AM	24	3.018	11.105	6.885	1.979	9.031	4.435	4.379	1.857	-0.643 (0.13)	-0.296 (0.17)
SIL	AF	24	3.174	33.864	7.243	0.825	3.283	1.845	1.727	0.643	0.078 (0.13)	0.247 (0.15)
SLI	AF	24	3.192	10.142	5.57	0.882	6.149	2.623	2.484	1.319	-0.26 (0.12)	-0.081 (0.14)
STE	J1M	24	3.008	8.884	5.465	0.433	2.14	1.124	1.117	0.422	0.555 (0.13)	0.316 (0.16)
STI	J1F	24	3.171	9.274	4.95	0.245	2.583	0.996	0.818	0.629	0.711 (0.14)	0.65 (0.18)
STR	J2M	24	2.985	9.164	5.596	0.983	4.479	1.953	1.485	0.969	0.106 (0.12)	0.052 (0.15)
TER	AF	24	4.262	17.583	8.864	2.345	10.766	5.433	4.702	2.238	-0.907 (0.13)	-0.772 (0.17)
THI	AF	24	3.035	8.541	5.794	1.235	3.517	1.956	1.838	0.565	0.035 (0.12)	0.208 (0.14)
TIL	J2F	24	2.989	9.17	5.371	0.202	5.666	1.392	1.095	1.063	0.571 (0.14)	0.351 (0.18)
TON	J1F	24	3.407	9.413	5.534	0.536	4.542	2.061	1.563	1.143	0.007 (0.14)	-0.064 (0.19)
TRI	AF	24	2.581	7.843	4.858	0.303	2.742	0.978	0.886	0.58	0.76 (0.14)	0.837 (0.15)
TRU	J1F	24	2.88	11.377	5.179	0.388	8.349	1.739	1.321	1.621	0.301 (0.13)	0.228 (0.18)
TUP	AF	24	3.051	8.863	5.724	1.228	5.29	2.825	2.725	0.992	-0.304 (0.13)	-0.153 (0.14)
YOD	J1F	24	3.048	11.576	5.887	1.913	7.601	3.906	3.334	1.538	-0.561 (0.12)	-0.62 (0.19)
YOL	AF	24	4.615	22.162	11.053	4.125	15.599	7.855	7.505	3.072	-1.357 (0.14)	-1.201 (0.18)

Text S5. Testing consistency of tolerance across years

To understand whether tolerance estimates varied between years we repeated the procedures outlined in [1] on a subset of 15 individuals (approximately 25% of group members) that were present across 2017, 2018, and 2019. Due to time constraints and results from the previous approach we allowed for up to 4 approaches per individual per sample day, but never sequentially. All individuals received 12 approaches by AA for the 2019 samples.

For the first part of this analysis, we combined the data collected during 2017 and 2018 with the data collected during 2019. The analysis described in [1] was repeated on this dataset. The only changes to this FID model were the removal of the observer identity and its interaction with trial number (per observer) as only AA completed trials in 2019 (observer identity had little effect on FID previously). Individual trial number per observer was included as a fixed effect and random slope over individual identity, trial number restarted from 1 for AA's additional trials in 2019 given the previous trials had taken place more than 12 months previously. Group trial number per day and individual trial number per day were also included as fixed effects to control for habituation and sensitization effects across a number of temporal levels. We also included 'year' as a fixed effect to explore consistency between years across all individuals. We removed the fixed effects of neighbour flee first and external factors within 5 minutes from the models used in [1] as they were previously shown not to effect FID in a significant way and we did not want to over parameterize the model. With 2017 as the reference category, the model estimates for 2018 and 2019 were 0.11 (-0.06,0.28) and 0.05 (-0.11,0.22) respectively. In each case estimates were close to zero with credible intervals overlapping zero, providing strong support for there being no effect of year on FID.

In addition, we ran a 2019 model using the same analytical framework as described above based only on 2019 FID data from the sample of 15 individuals. The only changes to the model was the removal of the covariate 'year'. We then extracted the individual conditional modes from the model and performed a Pearson's correlation between the 2019 conditional modes and the conditional modes from the updated FID model described in text S3 and table S3. Results supported that tolerance estimates were consistent across years ($r(13) = .906$, $p < .001$), as such we felt confident utilising the data collected during 2017/2018 for all individuals in the updated. Conditional modes extracted from the previous study (see [1]) were originally on the spectrum whereby highly tolerant animals had low/negative estimates and highly intolerant animals had high/positive estimates; therefore, tolerance was multiplied by minus 1 to reverse the scale for more logical inference in this study.
